# Epitope-engineered human hematopoietic stem cells are shielded from CD123-targeted immunotherapy

**DOI:** 10.1084/jem.20231235

**Published:** 2023-09-29

**Authors:** Romina Marone, Emmanuelle Landmann, Anna Devaux, Rosalba Lepore, Denis Seyres, Jessica Zuin, Thomas Burgold, Corinne Engdahl, Giuseppina Capoferri, Alessandro Dell’Aglio, Clément Larrue, Federico Simonetta, Julia Rositzka, Manuel Rhiel, Geoffroy Andrieux, Danielle N. Gallagher, Markus S. Schröder, Amélie Wiederkehr, Alessandro Sinopoli, Valentin Do Sacramento, Anna Haydn, Laura Garcia-Prat, Christopher Divsalar, Anna Camus, Liwen Xu, Lorenza Bordoli, Torsten Schwede, Matthew Porteus, Jérôme Tamburini, Jacob E. Corn, Toni Cathomen, Tatjana I. Cornu, Stefanie Urlinger, Lukas T. Jeker

**Affiliations:** 1 Department of Biomedicine, Basel University Hospital and University of Basel, Basel, Switzerland; 2 Transplantation Immunology and Nephrology, Basel University Hospital, Basel, Switzerland; 3 Cimeio Therapeutics AG, Basel, Switzerland; 4 Ridgeline Discovery GmbH, Basel, Switzerland; 5 Translational Research Centre in Onco-Hematology, Faculty of Medicine, University of Geneva, and Swiss Cancer Center Leman, Geneva, Switzerland; 6 Division of Hematology, Department of Oncology, Geneva University Hospitals, Geneva, Switzerland; 7 Department of Medicine, Translational Research Center for Onco-Hematology, Faculty of Medicine, University of Geneva, Geneva, Switzerland; 8 Institute for Transfusion Medicine and Gene Therapy, Medical Center - University of Freiburg, Freiburg, Germany; 9 Center for Chronic Immunodeficiency, Faculty of Medicine, University of Freiburg, Freiburg, Germany; 10 Institute of Medical Bioinformatics and Systems Medicine, Medical Center-University of Freiburg, Freiburg, Germany; 11 Department of Biology, Institute of Molecular Health Sciences, ETH Zürich, Zürich, Switzerland; 12 Department of Pediatrics, School of Medicine, Stanford University, Stanford, CA, USA; 13 Biozentrum, University of Basel, Basel, Switzerland; 14 SIB Swiss Institute of Bioinformatics, Basel, Switzerland

## Abstract

Targeted eradication of transformed or otherwise dysregulated cells using monoclonal antibodies (mAb), antibody–drug conjugates (ADC), T cell engagers (TCE), or chimeric antigen receptor (CAR) cells is very effective for hematologic diseases. Unlike the breakthrough progress achieved for B cell malignancies, there is a pressing need to find suitable antigens for myeloid malignancies. CD123, the interleukin-3 (IL-3) receptor alpha-chain, is highly expressed in various hematological malignancies, including acute myeloid leukemia (AML). However, shared CD123 expression on healthy hematopoietic stem and progenitor cells (HSPCs) bears the risk for myelotoxicity. We demonstrate that epitope-engineered HSPCs were shielded from CD123-targeted immunotherapy but remained functional, while CD123-deficient HSPCs displayed a competitive disadvantage. Transplantation of genome-edited HSPCs could enable tumor-selective targeted immunotherapy while rebuilding a fully functional hematopoietic system. We envision that this approach is broadly applicable to other targets and cells, could render hitherto undruggable targets accessible to immunotherapy, and will allow continued posttransplant therapy, for instance, to treat minimal residual disease (MRD).

## Teaser

This work demonstrates that engineering a single amino acid substitution into the cell surface protein CD123 in hematopoietic stem cells protects them from targeted immunotherapies. Transplantation of the modified stem cells may enable posttransplant therapy to reduce disease recurrence.

## Graphical Abstract

**Figure:**
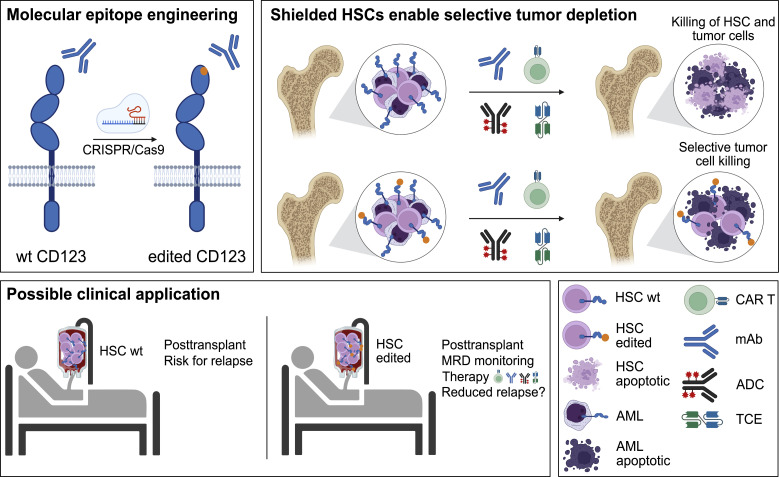

## Introduction

Targeted cell depletion represents a medical standard of care for several hematologic malignancies, autoimmune diseases, and prevention or treatment of acute rejection in organ transplantation. Depleting antibodies are mostly IgG, but other highly effective targeted immunotherapies work through different modes of action and include various antibody-derived molecular formats such as antibody–drug conjugates (ADC), radioimmunoconjugates, T cell engagers (TCE), or chimeric antigen receptor (CAR)–bearing cells (Carter and Rajpal, 2022; Globerson Levin et al., 2021; June et al., 2018). In the past decade, the latter has emerged as a highly effective, programmable cell depletion modality with very high response rates in B cell malignancies and more recently systemic lupus erythematosus (Brentjens et al., 2011; Kalos et al., 2011; Kochenderfer et al., 2010; Lee et al., 2015; Mougiakakos et al., 2021; Porter et al., 2011). The persistence of CD19 CAR T cells can be reliably measured by assessing the duration of B cell aplasia after infusion (Finck et al., 2022; Kochenderfer et al., 2010; Paszkiewicz et al., 2016). In children and young adults with B cell acute lymphocytic leukemia, it appears that persistence of CAR T cells is an important requirement for cure (Majzner and Mackall, 2019). Hence, various strategies to increase CAR T longevity are explored (Eyquem et al., 2017; Lynn et al., 2019). Although high depletion efficiencies can lead to long-term remission, they are associated with the risk for prolonged B cell aplasia due to indiscriminate killing of both normal and tumor B cells, respectively (Maude et al., 2014; Porter et al., 2011). In fact, CAR T cells can persist for a decade and result in years-long B cell aplasia (Melenhorst et al., 2022). Such deep purging of a cell type is only acceptable if the depleted cell is dispensable and/or its function can be replaced. Thus, a key to the CAR T field’s success was the availability of B cell–restricted target antigens (e.g., CD19) and the possibility to mitigate the loss of co-targeted healthy B cells through infusions of immunoglobulins. Given the clinical benefit and the commercial availability of CAR T cells as well as other effective cell depleting modalities, the number of patients at risk for iatrogenic long-term immunodeficiencies through highly efficacious cell-depleting therapies are expected to continue to increase rapidly.

Unlike the breakthrough progress achieved for B cell malignancies, there is a pressing need to find suitable antigens for immunotherapy of myeloid malignancies, particularly acute myeloid leukemia (AML; Perna et al., 2017). Targeting myeloid malignancies is especially challenging however, since AML displays high clonal heterogeneity, being composed of cells with highly variable surface protein expression (Baroni et al., 2020; Haubner et al., 2019; Majzner and Mackall, 2019; Perna et al., 2017). Importantly, leukemia stem cells (LSCs), which are phenotypically very similar to HSCs, are important targets in AML (Zeng et al., 2022). Therefore, most AML candidate targets are co-expressed by hematopoietic stem and progenitor cells (HSPCs; Haubner et al., 2019; Perna et al., 2017). As a consequence, the risk of myelosuppression associated with myeloid cell–targeted CAR T therapy likely limited the number of clinical trials for AML compared with B cell targeted CAR T trials (Gill et al., 2014; Majzner and Mackall, 2019). Despite major efforts, single targets with an expression profile as favorable as CD19 could not be identified in AML (Perna et al., 2017). Therefore, the paucity or mere absence of cancer-restricted surface proteins constitutes a critical barrier to antigen-specific immunotherapy (Finck et al., 2022; Irving et al., 2022; Majzner and Mackall, 2019).

A number of solutions have been proposed to overcome the limitations of shared target antigens that underly on-target off-tumor toxicity. Affinity tuning, i.e., reducing a CAR’s affinity, can increase the selectivity toward cells with a high antigen expression (Vander Mause et al., 2022); transient CAR expression (delivery as mRNA) temporally limits CAR activity; and CAR T therapies targeting essential cells, particularly HSPCs, could be used as a bridge to transplant (Arai et al., 2018; Gill et al., 2014; Myburgh et al., 2020). However, sparing cells with low antigen expression creates a risk for antigen^−/lo^ relapse (Majzner and Mackall, 2018, 2019), and the need to remove an effective CAR T therapy targeting HSPCs is neither economically nor scientifically appealing. Therefore, three groups proposed a radical solution: removing the target antigen partially or entirely in HSPCs before hematopoietic stem cell (HSC) transplantation (HSCT) prevents binding of the immunotherapy to the HSPCs and their progeny and thus creates a synthetic tumor selectivity (Borot et al., 2019; Humbert et al., 2019; Kim et al., 2018). Preclinical studies demonstrate the feasibility for CD33, and early results from a clinical trial showed a favorable safety profile with engraftment of CD33-deficient CD34^+^ HSPCs (clinicaltrial.gov NCT04849910; Borot et al., 2019; Humbert et al., 2019; Kim et al., 2018). Additional data will be required for further development of this approach. However, the number of truly dispensable antigens is likely limited yet mostly unknown. Moreover, targeting dispensable proteins may favour antigen-negative cancer relapses, a phenomenon known to limit long-term outcome of CAR T therapies (Majzner and Mackall, 2018). Therefore, deliberately targeting essential proteins would be preferrable to reduce the risk for antigen escape. However, this is impossible with a knock-out (KO) approach.

Here, we aimed to provide proof-of-concept by engineering HSPCs expressing an endogenous protein variant that completely shields the cells from targeted immunotherapy while preserving its function. The IL-3 receptor α chain (*IL3RA*; CD123) regulates the proliferation and differentiation of HSPCs (Emerson et al., 1988), is often expressed on AML LSCs and blasts from relapses (Jordan et al., 2000), and hence is associated with a poor outcome of the disease. Therefore, CD123 constitutes a promising target for AML (Baroni et al., 2020; Gill et al., 2014) for which multiple therapeutics are being explored preclinically and clinically including IL-3 bound to diphtheria toxin (Liu et al., 2004), monoclonal antibodies (mAb) blocking IL-3 (Roberts et al., 2010) or engineered for enhanced antibody-dependent cellular cytotoxicity (ADCC; Busfield et al., 2014), an ADC (Kovtun et al., 2018), and bispecific TCEs, e.g., a CSL362/OKT3-TCE (Chichili et al., 2015; Hutmacher et al., 2019). Despite the promising target toxicity, many of these therapies displayed cytotoxicity toward HSPCs, monocytes, basophils, and plasmacytoid dendritic cells (pDCs; Baroni et al., 2020; Chichili et al., 2015; Gill et al., 2014). Thus, protecting HSPCs from immunotherapies is desirable, but due to CD123’s role in HSPC biology, it is unknown whether a CD123 KO approach would be viable or if a KO approach may result in impaired immune function. We identified multiple single amino acid substitutions that shielded from ADCC, ADC, TCE, and CAR T cell killing while preserving CD123 function. These variants enabled selective TCE- or CAR T–mediated tumor killing while engineered HSPCs were unaffected. CD123 KO HSPCs had a major competitive disadvantage in vitro while engineered CD123 knock-in (ki) cells were comparable with wild type (wt). We envision that this approach is broadly applicable, could render undruggable targets accessible to immunotherapy and may enable continued, posttransplant immunotherapy, for instance to treat minimal residual disease.

## Results

### Rational design of human CD123 protein variants to shield from targeted immunotherapy

**Figure 1.**
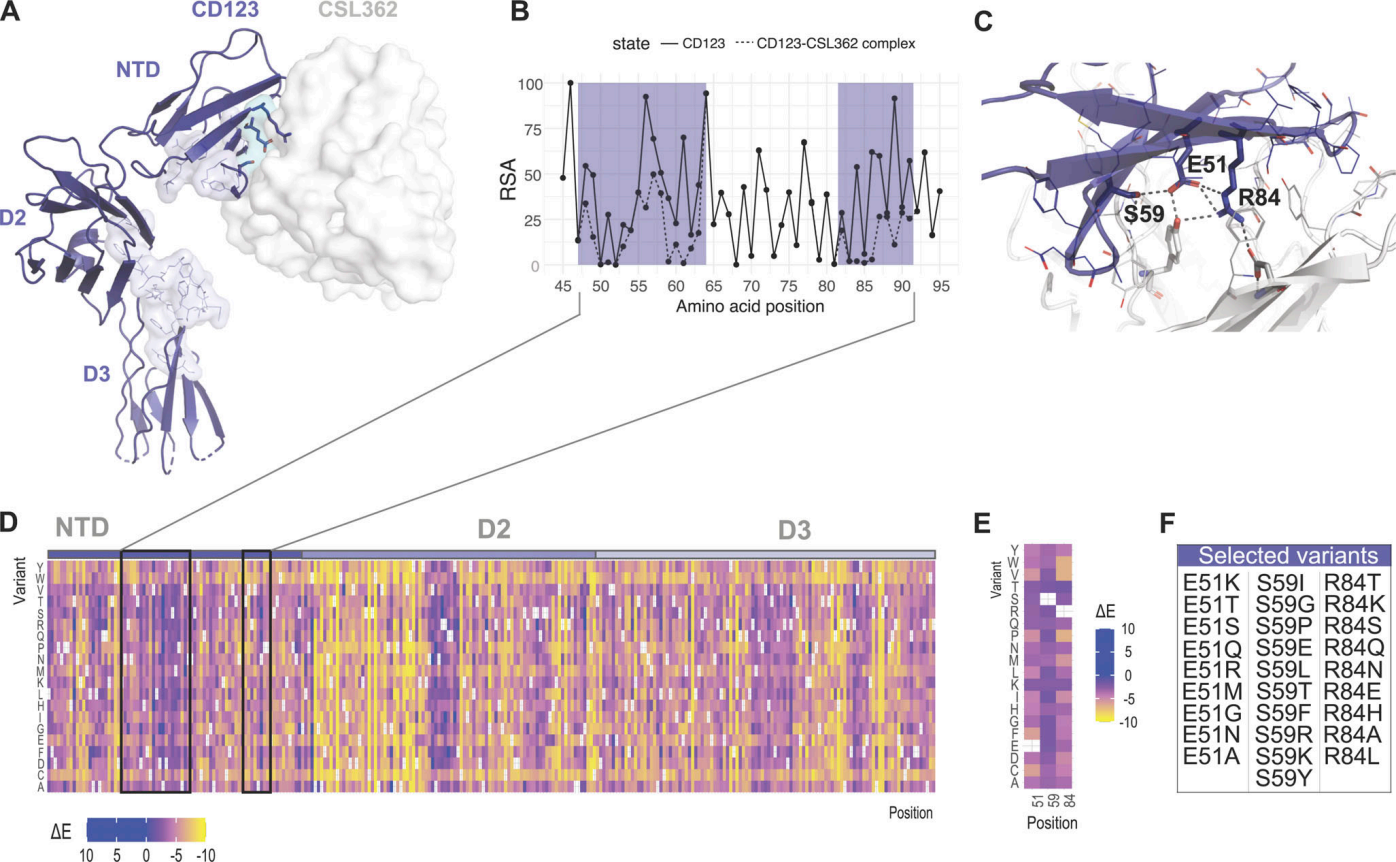
**Rational design of human CD123 protein variants to shield from targeted immunotherapy. (A)** Crystal structure of the CD123-CSL362 complex in open conformation (PDB ID: 4JZJ). CD123 is shown as ribbons. The CSL362 antibody variable domain is shown as a white surface. CD123 amino acid residues involved in IL-3 binding are highlighted as lines and in light blue. **(B)** Per-residue relative solvent accessibility (RSA) computed on the CSL362-free (solid line) and CSL362-bound (dashed line) states based on the x-ray structure of the CD123–CSL362 complex (PDB ID: 4JZJ). RSA data are shown for the NTD. The CSL362 epitope region is highlighted in blue. **(C)** Amino acid residues at the interface of the CD123–CSL362 complex are highlighted as lines and sticks. Side chain–mediated intermolecular contacts are shown as dashed black lines. **(D and E)** The predicted ΔE mutational landscape of CD123 is shown as a heatmap for the full ECD (residue range 20–305, x axis; D) and selected amino acid positions: E51, S59, and R84 (E). Heatmap color ranges from yellow (ΔE < 0, predicted damaging) to blue (ΔE ≥ 0, predicted neutral or beneficial). **(F)** Selected amino acid variants at residues E51, S59, and R84 are sorted by decreasing ΔE values.

CD123 variants were designed in silico. We aimed to identify protein variants that are structurally and functionally tolerated, i.e., variants that exhibit a similar structure to wt CD123, that preserve the ability to bind IL-3 and elicit IL-3–mediated downstream cell signaling but are otherwise shielded from the CSL362 antibody or a similar antigen binding moiety. To this aim, available experimentally determined three-dimensional structures of CD123 in different states were used to identify the protein regions involved in binding to CSL362 and IL-3. As previously described (Broughton et al., 2014), the CSL362 epitope is located at the N-terminal domain (NTD) of CD123 (Fig. 1 A), where the CSL362 fragment antigen binding binds both the “open” and “closed” conformations of the NTD. Based on structural analysis, we identified several CD123 residues as part of the antibody–antigen interface and involved in direct intermolecular interactions with the CSL362 complementarity-determining regions in both conformational states: T48, D49, E51, A56, D57, Y58, S59, M60, P61, R84, V85, A86, N87, P89, F90, and S91. Most of these amino acid sites show differential exposure to solvent upon binding of the CSL362 antibody, with residues E51, S59, P61, and R84 switching from highly exposed to buried (Fig. 1 B). E51, S59, and R84 were selected as critical sites for the CSL362 binding (Fig. 1 C) and most likely safe for mutagenesis. In contrast, P61 is likely relevant for IL-3 binding due to its close interatomic distance to IL-3 residues and therefore was excluded from further analysis (Broughton et al., 2014). For each position, comprehensive mutagenesis was performed in silico and the statistical energy difference (ΔE) was calculated to estimate the impact of the changes on CD123’s stability and function (Fig. 1 D). Taking into account the estimated mutational effect with particular focus on the three key residues E51, S59, and R84 (Fig. 1 E), we selected 28 candidate variants based on the ranking of ΔE values (Fig. 1 F).

### Preserved expression of engineered CD123 variants despite abolished binding to the mAb MIRG123

**Figure 2.**
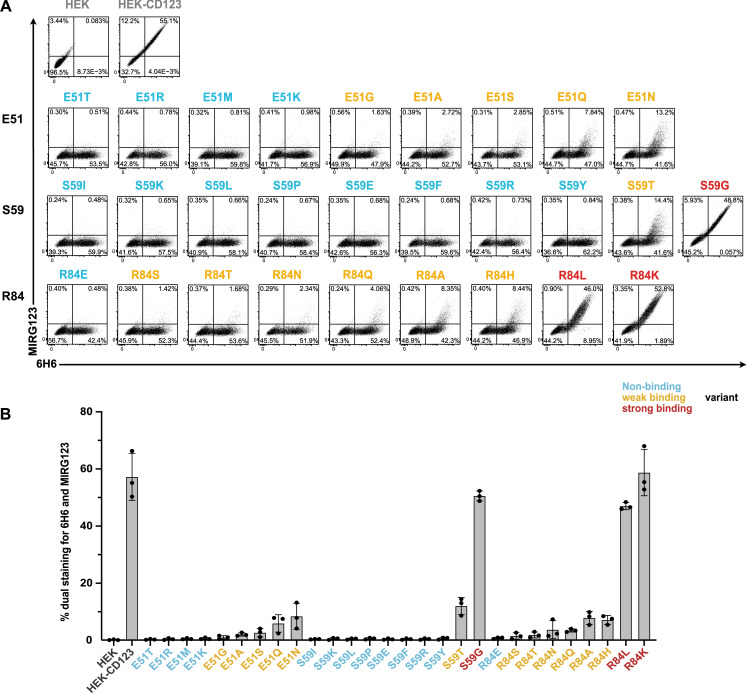
**Preserved expression of engineered CD123 variants despite abolished binding to the mAb MIRG123. (A and B)** Flow cytometry plots (A) and summarizing bar graph (B) showing binding of the anti-human CD123 antibody MIRG123 (biosimilar of CSL362) and the control clone 6H6 to wt CD123 and its 28 variants stably expressed in HEK-293 cells. Variants were categorized based on the dual staining to MIRG123 and 6H6 as non-binding (blue, <1% dual staining), weak (orange, 1–20%), or strong (red, >20%) binding variants. Control conditions (gray) are HEK-293 cells stably expressing wt CD123 (HEK-CD123) and non-transduced HEK-293 cells (HEK). Error bars: mean (SD). Data in A are representative of three independent experiments summarized in B.

We used a CSL362 IgG1 biosimilar (MIRG123) to experimentally validate its binding to the in silico–designed CD123 variants. We generated HEK-293 cells stably expressing human wt CD123 (HEK-CD123) or individually harboring each of the 28 selected amino acid substitutions at positions E51, S59, and R84 (Fig. 1 F). Using flow cytometry, we concomitantly quantified MIRG123 binding as well as preserved expression of the variants by staining with the control anti-CD123 mAb clone 6H6, whose binding to CD123 does not interfere with MIRG123 (Fig. 2 A). The results revealed a drastic reduction of MIRG123 binding to most candidate CD123 variants and abolished binding to almost half (13/28) of them. Based on the dual staining characteristics of mAbs 6H6 and MIRG123, each variant was categorized as either a non-binding (<1% double staining to 6H6/MIRG123, 13 variants, blue), weak (1–20%, 12 variants, orange), or strong (>20%, 3 variants, red) binding variant (Fig. 2 B). The latter showed comparable binding to HEK-CD123. Among the weak binding variants, MIRG123 mostly bound the highest CD123-expressing cells. These results demonstrate the feasibility of rationally designing single amino acid substitutions that completely disrupt the binding of a candidate antibody while preserving the expression of the engineered protein.

### Cells expressing engineered CD123 variants are shielded from multiple targeted immunotherapy modalities in vitro

**Figure 3.**
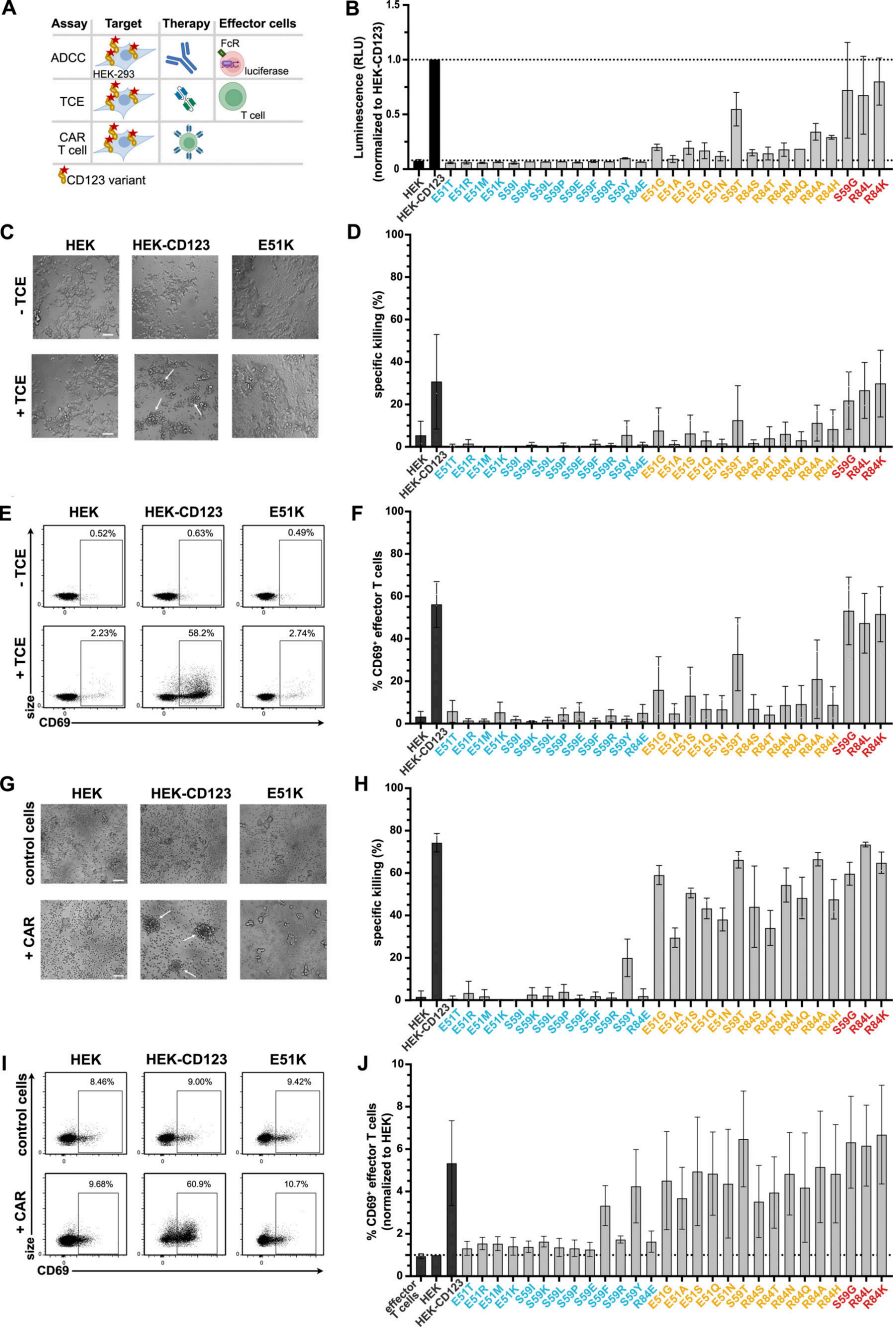
**Cells expressing engineered CD123 variants are shielded from multiple targeted immunotherapy modalities in vitro. (A)** Schematic to assess shielding of CD123 expressing cells from three targeted immunotherapies (ADCC, TCE, CAR T cell) in vitro. **(B)** MIRG123-induced ADCC measured by the luminescence of the effector cell line Jurkat/FcγRIIIa/NFAT-Luc following co-culture with HEK, HEK-CD123, or the CD123 variants. The luminescence signal normalized to the culture with HEK-CD123 (top dashed line). Data of two independent experiments. **(C–F)** 3-d co-culture of effector T cells and HEK-293 expressing CD123 and its variants with and without CSL362/OKT3-TCE (TCE). Data represent five independent donors and experiments with two technical replicates per group. **(C)** Representative images after 3-d co-culture with HEK, HEK-CD123, or E51K with and without TCE. White arrows indicate cell clustering. Scale bar: 100 μm. **(D)** Specific TCE-mediated killing of HEK-293 cells or its variants. **(E)** Representative flow cytometry plots indicating CD69-expression in effector T cells without (top) and with (bottom) TCE after 3 d co-cultures with HEK, HEK-CD123, and the variant E51K. **(F)** Frequency of CD69-expressing CD3^+^ effector T cells after 3 d. **(G–J)** Human 123CAR T cells were co-cultured with the target cells HEK, HEK-CD123, or its variants for 24 h. Control T cells were electroporated with an HDRT, Cas9 protein but no gRNA. Data are from three independent donors, each with two technical replicates. **(G)** Representative microscopy images after 1 d with white arrows indicating cell clustering. Scale bar: 100 μm. **(H)** Specific killing of target cells measured by flow cytometry at day 1 of co-culture. **(I and J)** Representative FACS plots (I) and (J) summary of CD69^+^ 123CAR T cells either alone (effector T cells) or in the presence of HEK, HEK-CD123, or all CD123 variants after 24 h co-culture. The data are normalized to % CD69^+^ cells in the presence of HEK target cells. **(B–J)** Error bars: mean (SD).

Next, we explored whether the non-binding variants were protected from MIRG123-mediated cytotoxicity either in the format of a mAb (ADCC), a bispecific TCE, or human CAR T cells (Fig. 3 A). First, we tested whether the CD123 variants were shielded from MIRG123-triggered ADCC using a FcγRIIIa-expressing reporter cell line. Target cell binding of the test antibody leads to the activation of a luminescence signal in the reporter cells through Fc-mediated activation of the FcγRIIIa receptor. High luminescence was measured in the presence of unmodified antigen (HEK-CD123) as well as all strong binding variants (Fig. 3 B). In contrast and consistent with the flow cytometry data (Fig. 2, A and B), 12/13 non-binding variants did not induce any ADCC signal above background detected in control cells devoid of CD123 (HEK; lower dashed line) with the exception of S59Y, which induced minimal activity. Weak binders showed an intermediate ADCC activity. Thus, ADCC signals positively correlated with the flow cytometry data.

**Figure S1.**
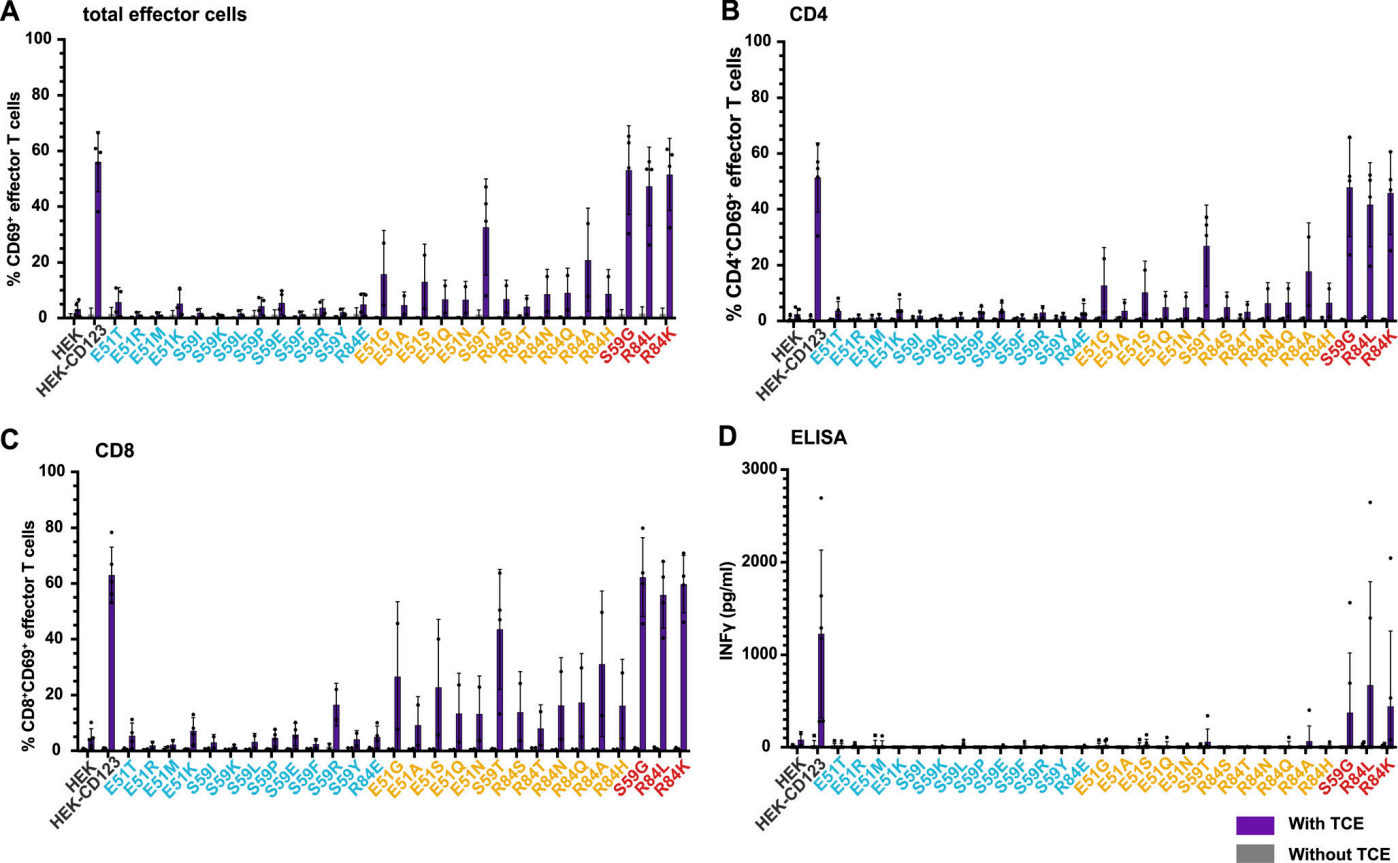
**TCE-mediated effector T cell activation. (A–D)** 72 h co-culture of human effector T cells with HEK, HEK-CD123, and CD123 variants (effector-to-target ratio = 10:1) in the presence of the CSL362/OKT3-TCE (300 ng/ml). Percentage of CD69^+^ cells within total (A), gated CD4^+^ (B), and CD8^+^ (C) effector T cells with (purple) and without (gray) TCE. Data are from five independent donors and experiments with two technical replicates per group. **(D)** IFNγ secretion was measured by ELISA in co-culture supernatants after 72 h. **(A–D)** Data are from four blood donors and experiments with two technical replicates per group. Error bars: mean (SD).

Encouraged by these results, we investigated whether the non-binding variants would also protect from more potent immunotherapies such as TCEs and CAR T cells which result in cytotoxicity at a lower antigen density than ADCC-inducing mAbs (Strohl and Naso, 2019). First, we co-cultured CD123 expressing HEK-293 cells and human T cells with and without the CSL362/OKT3-TCE (TCE; Hutmacher et al., 2019). After 3 d of co-culture, T cells clustered in the presence of HEK-CD123 and TCE, whereas clusters were neither seen with the non-binding variant E51K (or any of the other non-binding variants) nor with the target cells devoid of CD123 (HEK; Fig. 3 C). In line with these findings, neither HEK cells nor cell lines expressing non-binding CD123 variants were subject to TCE-mediated cytotoxicity. In contrast, some weak-binding CD123 variants induced limited cytotoxicity while control HEK-CD123 and strong-binding variants were lysed in the presence of the TCE (Fig. 3 D). Consistent with these results, upregulation of the activation marker CD69 above background was detected in T cells in co-culture with HEK-CD123 but not with E51K or HEK (Fig. 3 E) or any of the non-binding CD123 variants (Fig. 3 F and Fig. S1 A). Of note, the presence of the TCE itself induced a minor CD69 upregulation by almost 3% above the background (Fig. 3 E). In contrast, the frequency of CD69^+^ T cells increased in the presence of some weak binding variants and was comparable between HEK-CD123 and the strong binding variants. Both CD4^+^ and CD8^+^ T cells were activated but CD8^+^ T cells displayed a higher proportion of activated CD8^+^CD69^+^ effector T cells (Fig. S1, B and C). Likewise, secreted interferon γ (IFNγ) was only detected in the co-culture supernatants from highly activated T cells triggered by strong antigen binding (Fig. S1 D).

**Figure S2.**
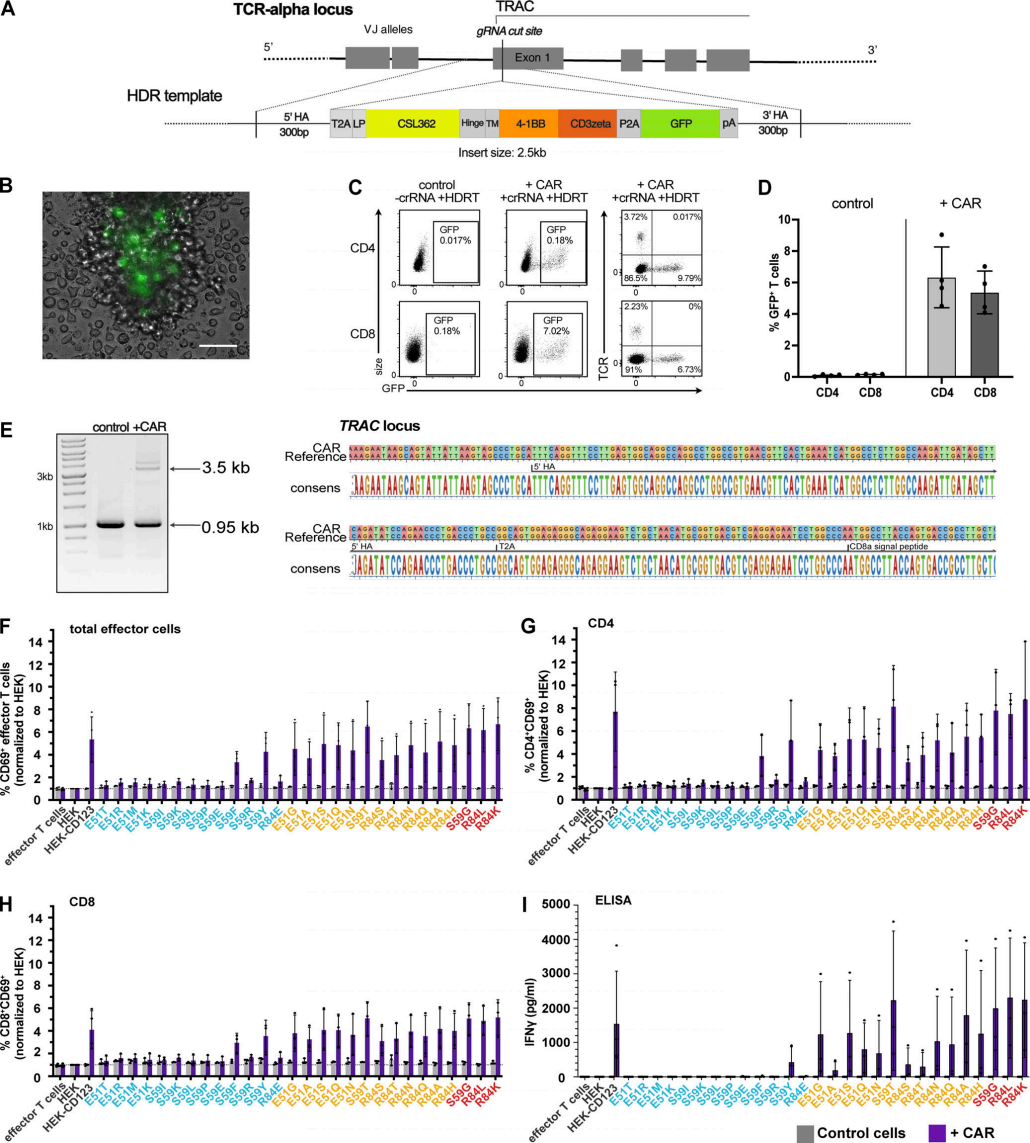
**123CAR design, production, and function. (A)** Non-viral HDR-mediated integration of the CD123-specific second-generation CAR into Exon 1 of the TRAC locus using CRISPR/Cas9. **(B)** Representative microscopy image day 4 after EP showing GFP^+^ cells expressing the CAR-encoding template. Scale bar 100 μm. **(C)** Flow cytometry plots highlighting CAR insertion into the TRAC locus represented by fluorescent intensity of GFP with disrupted endogenous TCR expression in human CD4^+^ and CD8^+^ T cell subsets. **(D)** Mean ki efficiency of the CAR-encoding template in gated CD4^+^ and CD8^+^ T cells at day 4–5. Control indicates cells electroporated with the HDRT, but incomplete RNPs. Data are from four independent donors and experiments. **(E)** Sanger sequencing results (left: gel image; and right: sequencing) confirm correct HDRT integration at the TRAC locus in flow-sorted GFP^+^ CAR cells with primers annealing outside both arms of homology. **(F–I)** 123CAR T cells (purple) or control cells (gray) were co-cultured with HEK, HEK-CD123, or its variants at an effector-to-target ratio of 10:1 for 24 h. Summary of flow cytometry data indicates the percentage of CD69^+^ cells within total (F), gated CD4^+^ (G), or CD8^+^ (H) control (gray), or 123CAR (purple) T cells after 24 h co-culture. **(I)** Quantification of IFNγ in supernatants of 24 h co-cultures using ELISA. Error bars: mean (SD). Data are from three independent donors and experiments with two technical replicates per group. Source data are available for this figure: SourceData FS2.

As a third modality of CD123-targeting immunotherapies, we investigated CAR T cells. We used non-viral clustered regularly interspaced short palindromic repeats (CRISPR)/Cas9-mediated homology-directed repair (HDR) to integrate a second-generation CSL362-derived CD123-specific CAR (123CAR) into the T cell receptor α constant region (*TRAC*; Fig. S2 A). Up to 9% of all electroporated CD4^+^ and CD8^+^ T cells expressed the 123CAR as assessed by GFP (Fig. S2, B–D). Correct 123CAR integration at the T cell receptor alpha constant region (*TRAC*) locus was confirmed by Sanger sequencing (Fig. S2 E). GFP^+^123CAR T cells were purified by flow cytometry, expanded, and subsequently used for in vitro cytotoxicity assays. CAR-less control T cells or 123CAR T cells were co-cultured for 24 h with the CD123 variant expressing target cell lines. Within hours, 123CAR T cells clustered around HEK-CD123 cells, whereas neither HEK nor E51K triggered 123CAR T clustering (Fig. 3 G). As observed for ADCC and TCE, 12/13 non-binding CD123 variants were shielded from cytotoxicity (Fig. 3 H). The only exception was again S59Y, which induced cytotoxicity (Fig. 3 H) and IFNγ secretion (Fig. S2 I). In contrast and different to the results observed for ADCC and TCE, both weak and strong binding variants triggered strong cytotoxicity (Fig. 3 H) and IFNγ secretion (Fig. S2 I). In accordance, neither HEK nor E51K increased the proportion of activated CD69^+^ 123CAR T cells above the background whereas, the frequency of CD69^+^ 123CAR increased in response to unmodified CD123 (Fig. 3 I). The exceptions were S59F and S59Y which both induced CD69 upregulation in CD4^+^ and CD8^+^ 123CAR T cells, respectively (Fig. 3 J and Fig. S2, F–H). However, among the non-binders, only S59Y concomitantly induced CD69 upregulation, IFNγ secretion, and cytotoxicity whereas S59F resulted in isolated CD69 upregulation but neither IFNγ secretion nor cytotoxicity. Thus, in contrast to ADCC and TCE where antibody and T cell effector function correlated well with antibody binding as determined by flow cytometry (Fig. 3, B–F; and Fig. 2 B), weak-binding CD123 variants induced 123CAR T responses comparable to strong-binding CD123 variants. These findings suggest that CAR T cells are more potent than ADCC and TCE, and even weak residual binding can result in CAR T activation and elimination of target cells. In summary, we demonstrate that a series of single amino acid substitutions in CD123 is sufficient to completely prevent the activity of an ADCC-inducing mAb, a TCE and a CAR T cell. This suggests that such variant proteins—when engineered into the genome of target cells—might provide protection from different targeted immunotherapies.

### Biophysical characterization of selected CD123 protein variants

**Figure 4.**
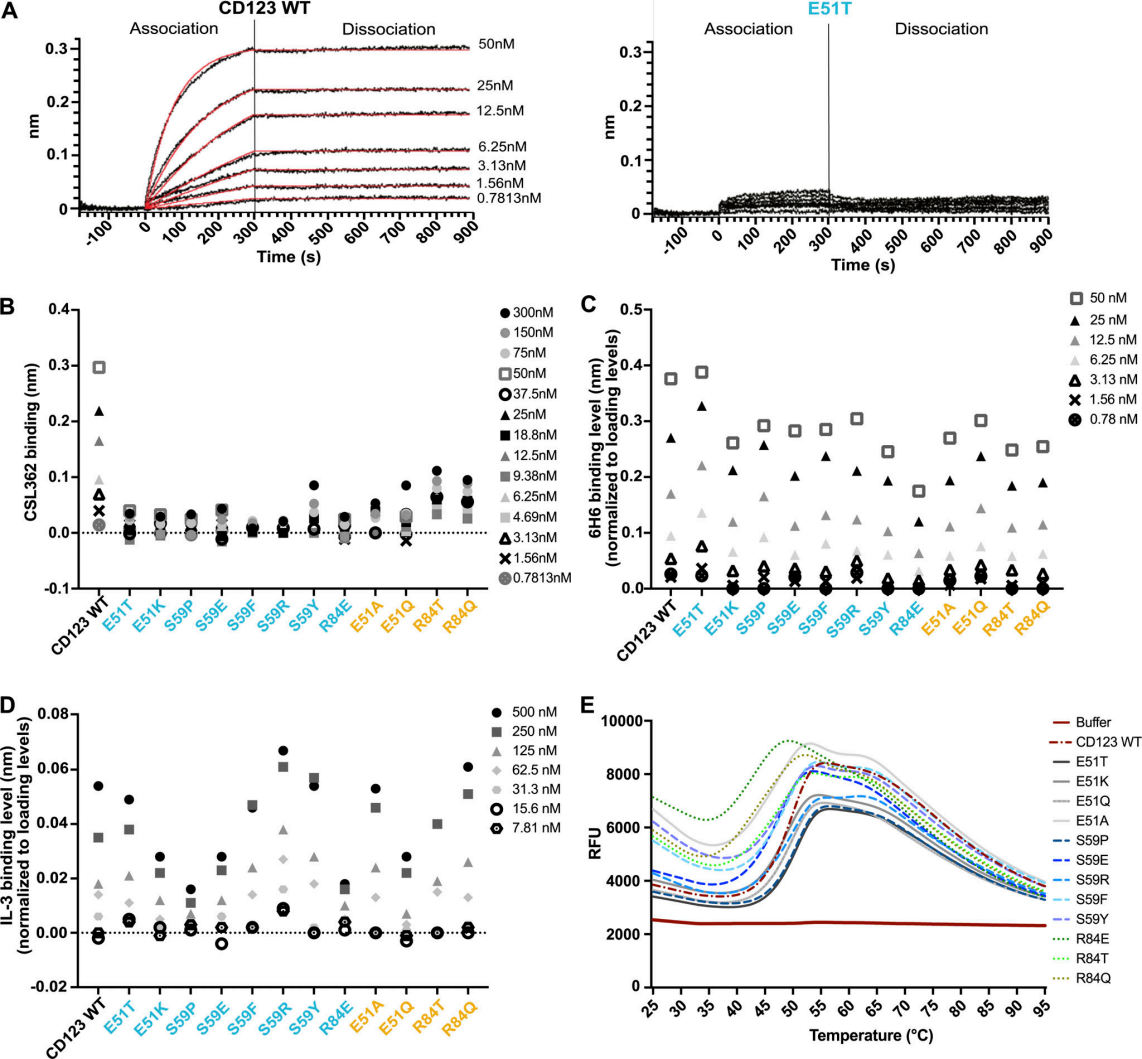
**Biophysical characterization of selected CD123 protein variants. (A)** Binding of CSL362 to the recombinant ECD of CD123 wt (left) and CD123 E51T (right) at increasing concentrations of CSL362 measured by BLI. **(B)** Binding levels of CD123 wt and its variants at different concentrations to captured CSL362 at 280 s. CD123 wt reaches its saturation to CSL362 at 50 nM, therefore higher concentrations were not measured. **(C)** Binding levels of CD123 wt and its variants to the captured antibody 6H6 (normalized to a loading level of 6H6) at 250 s. **(D)** Binding levels of IL-3 to biotinylated CD123 wt and variants (normalized to loading levels of biotinylated CD123 wt and its variants) at 250 s. **(E)** Thermal unfolding (relative fluorescence unit, RFU) of CD123 wt and variants measured by DSF with increasing temperatures. **(A–E)** Representative data are from two experiments with two technical replicates.

To characterize the effect of the introduced point mutations on the protein biophysical properties, the extracellular domains (ECD; amino acids 19–305) of selected CD123 variants were synthesized as soluble proteins. Real-time interaction with the immobilized CSL362 was measured using Bio-Layer Interferometry (BLI) at increasing concentrations of soluble CD123 ECD variants. wt CD123 is associated rapidly with the antibody CSL362 in a dose-dependent manner (Fig. 4 A). In contrast, no interaction was observed up to 300 nM of the soluble analyte CD123 E51T, a variant characterized as a non-binder by flow cytometry (Fig. 4 A and Fig. 2, A and B). Similarly, no association to CSL362 was observed for other non-binding variants, including E51K, S59P, S59E, S59F, S59R, and R84E. Residual CSL362 association was detected at different concentrations of the weak-binding variants E51A, E51Q, R84T, and R84Q (Fig. 4 B). Consistent with the shielding assays (Fig. 3, B–J), S59Y showed similar residual binding as weak-binders. Importantly, the association to the control antibody 6H6 was preserved in all tested variants, except for R84E (Fig. 4 C). Next, we assessed the functionality of the variants in terms of binding to IL-3, i.e., the physiologic ligand of CD123, to the immobilized CD123 variants. All tested variants bound to increasing concentrations of IL-3, with S59P and R84E displaying a modest reduction in IL-3 binding (Fig. 4 D). Lastly, protein thermal stability was assessed by differential scanning fluorimetry (DSF) analysis. Most CD123 variants demonstrated a thermostability comparable with CD123 wt with an unfolding temperature (Tm) of 50°C. In contrast, R84E induced a higher fluorescence signal at ambient temperature and featured a lower Tm (43°C; Fig. 4 E). Since R84E showed reduced binding to 6H6 and IL-3 and a lower thermal stability compared with wt, it was excluded from further experiments. In summary, we identified several protein variants harboring single amino acid substitutions at two different residues (E51T, E51K, S59E, and S59R) that entirely abolish binding to MIRG123 but preserve protein stability, binding to the control mAb 6H6 and almost completely maintaining binding to the receptor’s natural ligand IL-3.

### HSPCs expressing CD123 variants E51K and E51T are functional, differentiate normally in vitro, and display a good safety profile

**Figure S3.**
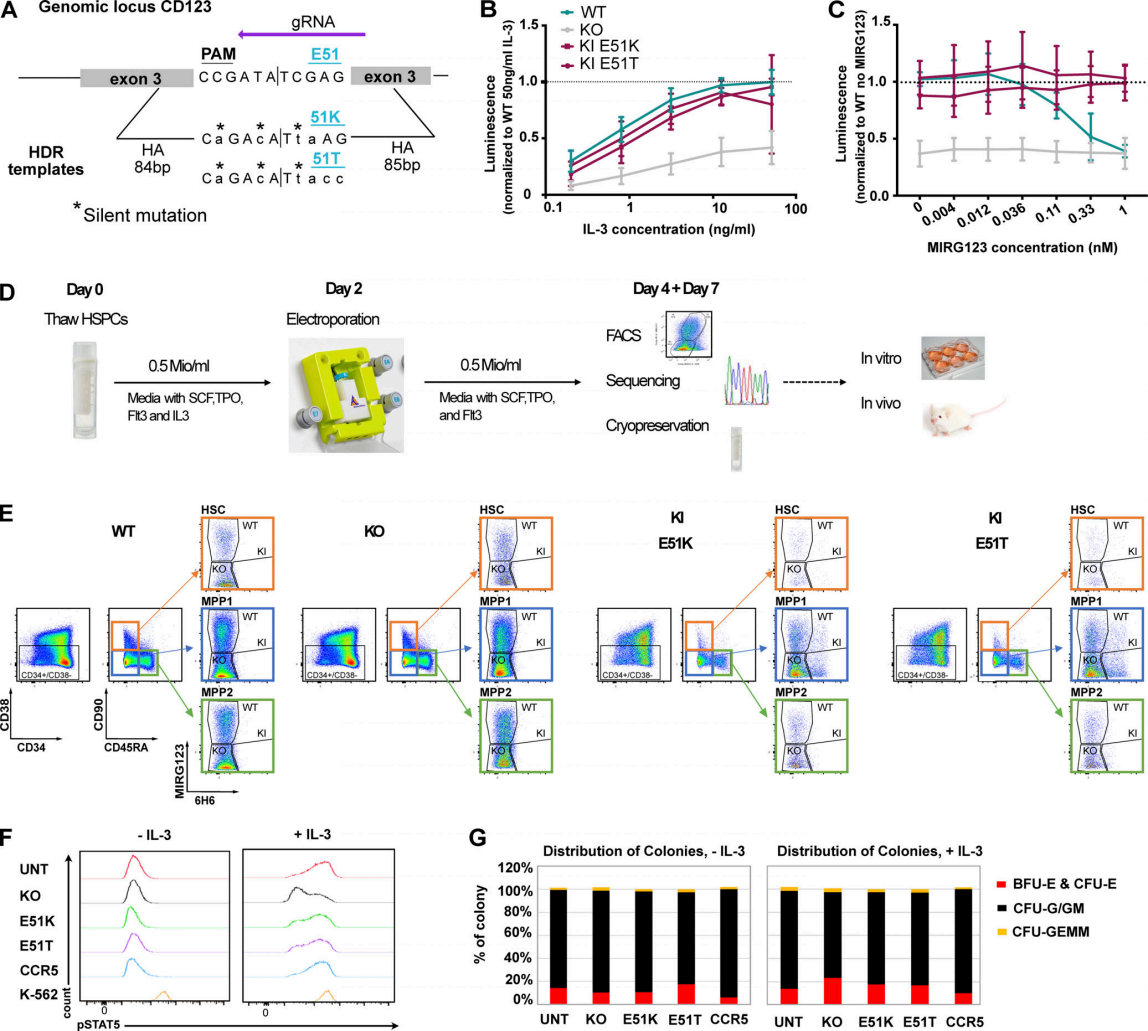
**TF-1 and HSPC epitope engineering. (A)** Schematic of HDRTs design for insertion of variants K and T at position 51. **(B and C)** Engineered TF-1 cells were sorted based on the binding to the anti-CD123 mAbs 6H6 and MIRG123 (wt MIRG123^+^6H6^+^, KO MIRG123^−^6H6^−^, and ki MIRG123^−^6H6^+^) and cultured for 3 d (B) with increasing concentrations of IL-3, or in C with 2.5 ng/ml IL-3 in the presence of increasing concentrations of MIRG123. Viable cells were quantified by luminescence and results were normalized to wt cells cultured with 50 ng/ml IL-3 (A) or to wt cells cultured without MIRG123 (B). Data are from four independent experiments. **(D)** Experimental design of non-viral CRISPR/Cas9-mediated HDR engineering of mobilized CD34^+^ enriched peripheral blood HSPCs using a GMP-compatible protocol with the CliniMACS Prodigy (Miltenyi). **(E)** Gating strategy to monitor CD123 expression in HSC (orange, CD34^+^CD38^−^CD90^+^CD45RA^−^), multipotent progenitor 1 (MPP1; blue; CD34^+^CD38^−^CD90^−^CD45RA^−^), and MPP2 (green; CD34^+^CD38^−^CD90^−^CD45RA^+^) using the mAbs MIRG123 and 6H6 2 and 5 d after EP. wt: MIRG123^+^6H6^+^, KO: MIRG123^−^6H6^−^, ki: MIRG123^−^6H6^+^ **(F)** Representative histogram of phosphorylated STAT5 upon exposure to IL-3 in AAV6-edited HSPCs. CCR5 KO was used as negative, and K-562 cells as positive control. Data represent two independent experiments. **(G)** In vitro differentiation of AAV6-edited HSPCs with and without IL-3. CFUs (erythroid: BFU-E & CFU-E, granulocytes/monocytes: CFU-G/GM, and myeloid progenitors: CFU-GEMM) were scored based on morphological characteristics. Data are from two independent experiments. Error bars: mean (SD).

Since we used a cell-free system to establish that the selected CD123 variants preserved dose-dependent binding of IL-3, we next aimed to investigate the functionality of E51K and E51T in intact cells. We used CRISPR/Cas9-mediated HDR to engineer the variants into the human erythroleukemia cell line TF-1 whose proliferation and survival is IL-3– or GM-CSF–dependent (Kitamura et al., 1989; Fig. S3 A). After cell sorting, the genetically engineered cells were cultured with increasing concentrations of IL-3. wt TF-1 displayed IL-3–dependent growth and the two ki populations E51K and E51T demonstrated near overlapping growth curves, indicating intact IL-3 sensing and signaling. In contrast, KO-sorted cells proliferated considerably less (Fig. S3 B). To test the blocking effect of MIRG123, we cultured the cells with a fixed IL-3 concentration but increasing amounts of MIRG123. As reported in a comparable system using BaF3 cells (Broughton et al., 2014), wt cells showed dose-dependent growth inhibition and died in the presence of antibody concentrations ≥0.036 nM. In contrast, E51K and E51T ki cells proliferated irrespective of the MIRG123 concentration. KO cells were not affected by MIRG123 but displayed decreased survival compared with wt, E51K, or E51T (Fig. S3 C).

**Figure 5.**
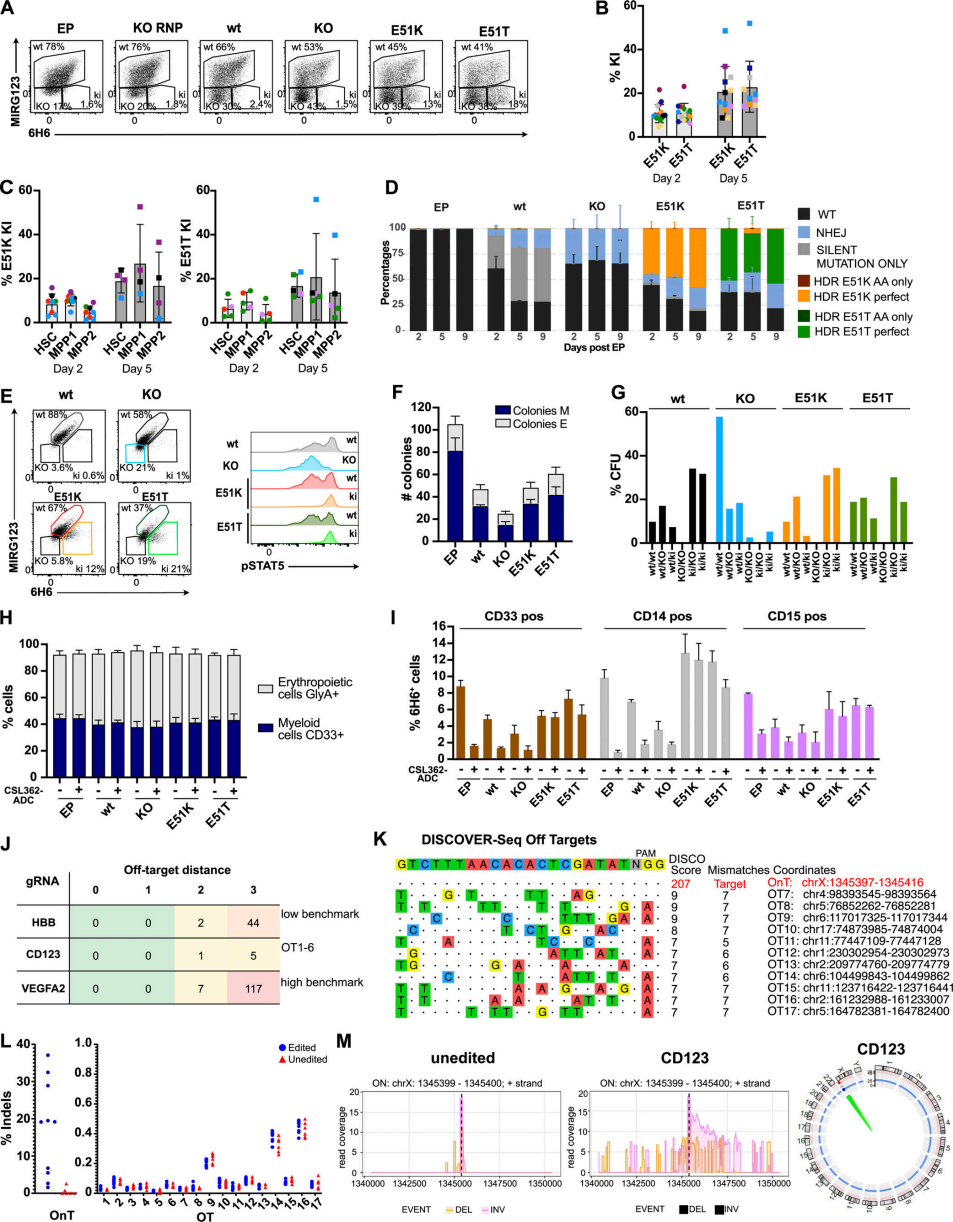
**HSPCs expressing CD123 variants E51K and E51T are functional, differentiate normally in vitro, and display a good safety profile. (A–M)** Characterization of non-virally CRISPR/Cas9-edited human CD34^+^ HSPCs. **(A)** Representative flow cytometry plots showing binding (%) of the anti-human CD123 antibody clones 6H6 and MIRG123 to edited CD34^+^ HSPCs 5 d after EP. EP (cells electroporated with Cas9 protein only); KO RNP (EP with RNP only); wt, KO, E51K, and E51T variants (electroporated with respective HDRT). In flow cytometry, cells double-positive for MIRG123 and 6H6 were defined as "wt," whereas MIRG123^−^6H6^−^ are indicated as “KO” although they include intended CD123 KO cells as well as cells naturally not expressing CD123. The MIRG123^−^6H6^+^ cell population is labeled as KI. Representative plots of five independent experiments each performed with different donors. **(B)** Frequency of ki cells (MIRG123^−^6H6^+^) 2 and 5 d after EP. Data from eight individual donors (each a color) were performed in six independent experiments with two to four technical replicates. **(C)** Quantification of the ki population in LT-HSCs (CD34^+^CD38^−^CD90^+^CD45RA^−^), multipotent progenitor 1 (MPP1; CD34^+^CD38^−^CD90^−^CD45RA^−^) and MPP2 (CD34^+^CD38^−^CD90^−^CD45RA^+^). **(D)** Representative Amplicon-NGS sequencing of the targeted CD123 locus at 2, 5, and 9 d after editing of control (EP), wt template, KO template, E51K, and E51T conditions. Data from four different experiments performed with different donors were pooled. **(E)** Representative FACS plots of CD123 stained with 6H6 and MIRG123 (left) and histograms of phosphorylated STAT5 (right) upon exposure to IL-3 in non-virally edited HSPCs. Color-coding in FACS plots and histogram is identical. Data are representative of four independent experiments. **(F)** In vitro differentiation of CD123-engineered HSPCs assessed by the number of colony-forming units (erythroid: E, myeloid: M). CFU were scored using STEMVision based on morphological characteristics. A representative experiment of two independent biological replicates performed in duplicates. **(G)** Allele frequency of the CD123-engineered HSPCs in a minimum of 38 colonies. **(H)** Frequency of GlyA^+^ and CD33^+^ non-virally edited HSPCs cultured in high cytokine medium with or without CSL362-ADC for 14 d. Myeloid lineage: CD33^+^, erythroid lineage: GlyA^+^. Data are from three experiments performed in triplicates. **(I)** Frequency of 6H6^+^ CD123-positive cells in the CD33^+^, CD14^+^, or CD15^+^ subsets. A representative experiment of two independent experiments performed in triplicates. **(J)** Computational off-target prediction. HBB and VEGFA site 2 as benchmarking gRNAs. On-target (OnT), off-target (OT1-6). **(K)** DISCOVER-Seq in KO RNP edited HSPCs. On-target (OnT), off-target (OT7-17). **(L)** rhAMPSeq validation of computational prediction and DISCOVER-Seq analysis. Shown are the editing rates as percentage of indels detected at the on-target (OnT) site and at the 17 off-target (OT) sites. Blue circles: Edited category comprises samples treated with gRNA for CD123 (KO RNP, KO template, E51K, and E51T). Red triangles: Unedited category comprises samples not treated with gRNA for CD123 (HSC, EP). Data are from one experiment performed with six samples generated in six independent editing experiments with cells from different donors. **(M)** CAST-Seq in unedited (left) and KO RNP edited HSPCs (center). Coverage plots of the on-target site in CD123 indicate large inversions (pink line) and deletions (orange line), respectively. Circos plot is used to illustrate chromosomal translocations. Data are from one experiment performed with two independent samples. Error bars: mean (SD).

Next, we aimed to engineer clinically relevant HSPCs that can potentially be used to provide a patient with a CD123 immunotherapy-resistant hematopoietic system. We used reagents close to good manufacturing practice (GMP)–grade with the goal of providing scientific proof of concept with the potential for clinical translation. To this end, we engineered the variants E51K and E51T into mobilized, CD34^+^ enriched peripheral blood HSPCs from healthy donors. CD34^+^ HSPCs were electroporated with high-fidelity SpCas9 ribonucleoproteins (RNPs) and single-strand oligodeoxynucleotides (ssODNs). CD123 expression was monitored using flow cytometry with the mAbs MIRG123 and 6H6, 2 and 5 d after electroporation (EP), respectively (Fig. 5 A and Fig. S3 D). Preliminary experiments demonstrated that the ssODNs reduced HSPC viability and consequently in vivo engraftment when E51K and E51T HSPCs were compared with HSPCs that were electroporated without an ssODN. Therefore, we designed ssODNs containing the same silent mutations as in E51K and E51T ssODNs (wt template), and a ssODN containing stop codons in all three reading frames (KO template) to have similar ssODNs as in the control samples. These groups were compared with E51K and E51T ki HSPCs as well as EP only and RNP without ssODN (KO). In comparison with electroporated wt cells (EP), cells receiving RNPs without ssODN displayed only a slightly increased fraction of MIRG123^−^6H6^−^ (KO) cells (Fig. 5 A). In contrast, HSPCs receiving RNP together with the wt template displayed increased CD123 KO, an effect that was further enhanced in the KO template group as has been reported before. The E51K or E51T HSPCs displayed a distinctly different FACS profile. Over time, a population with retained 6H6 but abolished MIRG123 binding (MIRG123^−^6H6^+^) gradually appeared (average 10% on day 2; average 21% on day 5 across eight donors), suggesting that these were E51K or E51T ki HSPCs (Fig. 5, A and B). Importantly, CD34^+^CD38^−^CD90^+^CD45RA^−^ cells (HSCs) displayed similar editing rates as the bulk CD34^+^ HSPCs, suggesting that long-term repopulating HSCs (LT-HSC) and multipotent progenitors MPP1 and MPP2 were edited at similar frequencies (Fig. 5 C and Fig. S3 E). Correct editing of E51 (GAG) to K (AAG) or T (ACC) was confirmed by next-generation amplicon sequencing (Amplicon-NGS; Fig. 5 D). The number ofki reads correlated well with the FACS analysis (Fig. 5, A, B, and D). To investigate whether the variants E51K and E51T preserved IL-3 signaling, we analyzed phospho-STAT5 (pSTAT5) in ki HSPCs and compared them with HSPCs engineered with wt template or KO template. Among CD123^+^ wt cells (MIRG123^+^6H6^+^ “wt,” left panel), IL-3 induced a bimodal pSTAT5 signal in both control HSPCs (wt template) as well as E51K and E51T ki HSPCs (Fig. 5 E, histograms). Within MIRG123^−^6H6^−^ CD123 KO cells (KO template), pSTAT5 signaling was abolished (Fig. 5 E, histogram). In contrast, gating on MIRG123^−^6H6^+^ (ki) cells demonstrated that all E51K and E51T ki cells were pSTAT5^+^. Thus, we leveraged the ability to discern unedited from edited cells by flow cytometry to demonstrate that all phenotypically defined ki cells transmitted the IL-3 signal. To further characterize the functionality of the engineered HSPCs and to determine the functional relevance of a preserved IL-3/CD123 signaling axis, we analyzed the HSPC in vitro differentiation potential in a colony-forming assay. Compared with HSPCs that were electroporated only (EP), the number of colonies was reduced in all samples electroporated with an ssODN template (Fig. 5 F). Among the latter, HSPCs electroporated with the KO template formed the lowest number of colonies. In contrast, E51K and E51T HSPCs formed equal (E51K) or slightly increased (E51T) number of colonies when compared with wt template HSPCs. However, the relative distribution of colonies representing different lineages (erythroid [E] and myeloid colonies [M]) was comparable among all genotypes (Fig. 5 F). These results suggested that CD123-deficient HSPCs could have a competitive disadvantage compared with wt or ki HSPCs. To verify this, we analyzed the allele frequency (genotypes) found in individual single cell-derived colonies and categorized the alleles as “wt” (unedited), “KO” (non-homologous DNA end joining), and “ki” (identification of ki template; Fig. 5 G). In colonies derived from “wt template” we found only paired alleles with the ability to express intact CD123, i.e., indels were only found when the other allele was wt or ki, and no KO/KO sequences were identified. In KO template HSPCs, only very few colonies harbored a genotype leading to the absence of CD123 (KO/KO, ki/ki or ki/KO), whereas the majority of colonies were unedited or contained at least one wt allele (wt/wt, wt/KO or wt/ki). Similarly, in E51K and E51T HSPCs, we did not find any KO/KO colonies. In contrast, ki/ki colonies were found at least with equal frequency when compared to wt/wt. Thus, there was a strong counterselection against CD123-deficient colonies whereas CD123 ki sequences were found at the expected frequencies. Therefore, the defect of CD123-deficient colonies in Fig. 5 F is an underestimate, and the real competitive disadvantage of CD123 KO HSPCs is much more pronounced. In contrast, CD123 E51K and E51T ki HSPCs are functionally equivalent to wt with regards to colony forming and differentiation potential. This was confirmed by a flow cytometry–based in vitro differentiation assay as all tested genotypes displayed an equal differentiation into CD33^+^ myeloid and GlycophorinA (GlyA)^+^ erythroid cells (Fig. 5 H). Of note, also in the presence of a CSL362-ADC, HSPCs differentiated normally in myeloid and erythroid lineages. Finally, we evaluated the resistance of myeloid CD33^+^, CD14^+^, or CD15^+^ cells derived from CD123-engineered HSPCs to CSL362-ADC. CD123 expressing 6H6^+^ cells were strongly depleted in EP, wt, and KO samples (Fig. 5 I), whereas E51K and E51T edited cells were largely resistant to CSL362-ADC killing.

As an orthogonal engineering approach, we used adeno-associated virus (AAV6)–mediated HDR. Consistent with the data from the non-virally engineered HSPCs, IL-3 induced a strong pSTAT5 signal in both control HSPCs (non-edited and CCR5 edited) as well as E51K and E51T ki HSPCs (Fig. S3 F; and related to Fig. 5 E). In contrast, CD123 KO cells displayed strongly reduced pSTAT5 signaling. As with the non-virally engineered HSPCs, the relative distribution of colonies representing different lineages (BFU-E and CFU-E, CFU-G/GM, and CFU-GEMM) was comparable among all genotypes independent of IL-3 (Fig. S3 G; and related to Fig. 5 H). Thus, E51K and E51T variants were efficiently engineered into HSPCs using two orthogonal approaches, resulting in functional CD123 receptor expression with intact IL-3 signaling and normal in vitro differentiation capacity, yet were shielded from a CD123-targeted ADC.

To assess the safety of the guide RNA (gRNA) used to engineer the E51K and E51T variants, we used computational prediction, Discover-Seq (Wienert et al., 2019), and CAST-Seq (Rhiel et al., 2023; Turchiano et al., 2021). We used Cas-OFFinder to identify potential off-target sites linked to NGG protospacer adjacent motif (PAM) sequences (Bae et al., 2014). Considering sites with a maximum of three mismatches or two mismatches with one DNA and/or RNA bulge, we identified six candidate off-target sites (OT1-6; Fig. 5 J and Table S1). Using these parameters, the gRNA targeting vascular endothelial growth factor A (VEGFA site 2) resulted in a high number of predicted off-targets as previously reported (high benchmark; Chaudhari et al., 2020). In contrast and in line with previous reports, the gRNA targeting the hemoglobin subunit beta (HBB) locus had a low number of predicted off-targets (low benchmark; DeWitt et al., 2016; Wienert et al., 2019). Compared with these benchmarking gRNAs, the gRNA targeting CD123 had very low numbers of predicted off-targets. Next, we performed Discover-Seq and obtained a typical on-target profile at a good sequencing depth resulting in a Disco score of 207. In contrast, all nominated off-target hits had a very low Disco score (≤9) and at least five mismatches. Since Disco scores highly correlate with eventual indel frequencies (Wienert et al., 2019), these results suggested that the nominated off-target hits were likely false positives (Fig. 5 K and Table S2, A and B). We selected the top 11 nominations for validation by RNase H-dependent amplification and sequencing (rhAmpSeq) and classified all other hits as false positives. Multiplexed amplicon next-generation sequencing (NGS) on the selected 18 loci (1 on target [OnT], 6 computational, 11 Discover-Seq) performed on unedited HSPC and EP (“unedited”) and KO template, KO RNP, E51K, and E51T samples (“edited”) from four different donors did not show any evidence of indels (Fig. 5 L). Finally, as an additional orthogonal assay, we performed CAST-Seq analysis, which identifies chromosomal translocations and on-target site aberrations and nominates off-target sites. An aliquot of the same samples used for Discover-Seq was kept in culture for 5 d after EP. In samples edited with RNP complexes, CAST-Seq revealed chromosomal aberrations at the on-target site, such as large deletions and inversions, whereas unedited samples only showcased background activity (Fig. 5 M, left panel). The CAST-Seq coverage plot spans a region of ∼10 kb. As shown in the plot, the highest activity is observed around the dotted line representing the target site. Indels in this region were quantified by rhAmpSeq (Fig. 5 L, left panel). The significance of these aberrations is generally difficult to predict. In this particular case, disruption of the CD123 locus by indels or by large chromosomal deletions/inversions will most likely have a negative effect on the growth of the affected cells, i.e., these cells are likely to disappear in vitro and in vivo (Fig. 5, F and G). At the same time, off-target mediated translocations could not be detected (Fig. 5 M, right panel), confirming the high specificity of the used CRISPR-Cas nucleases. Thus, the selected gRNA did not result in any detectable off-target activity in all the assays performed, demonstrating the safe engineering of HSPCs.

### CD123 epitope–engineered HSPCs engraft, differentiate normally, and possess long-term reconstitution potential in vivo

**Figure 6.**
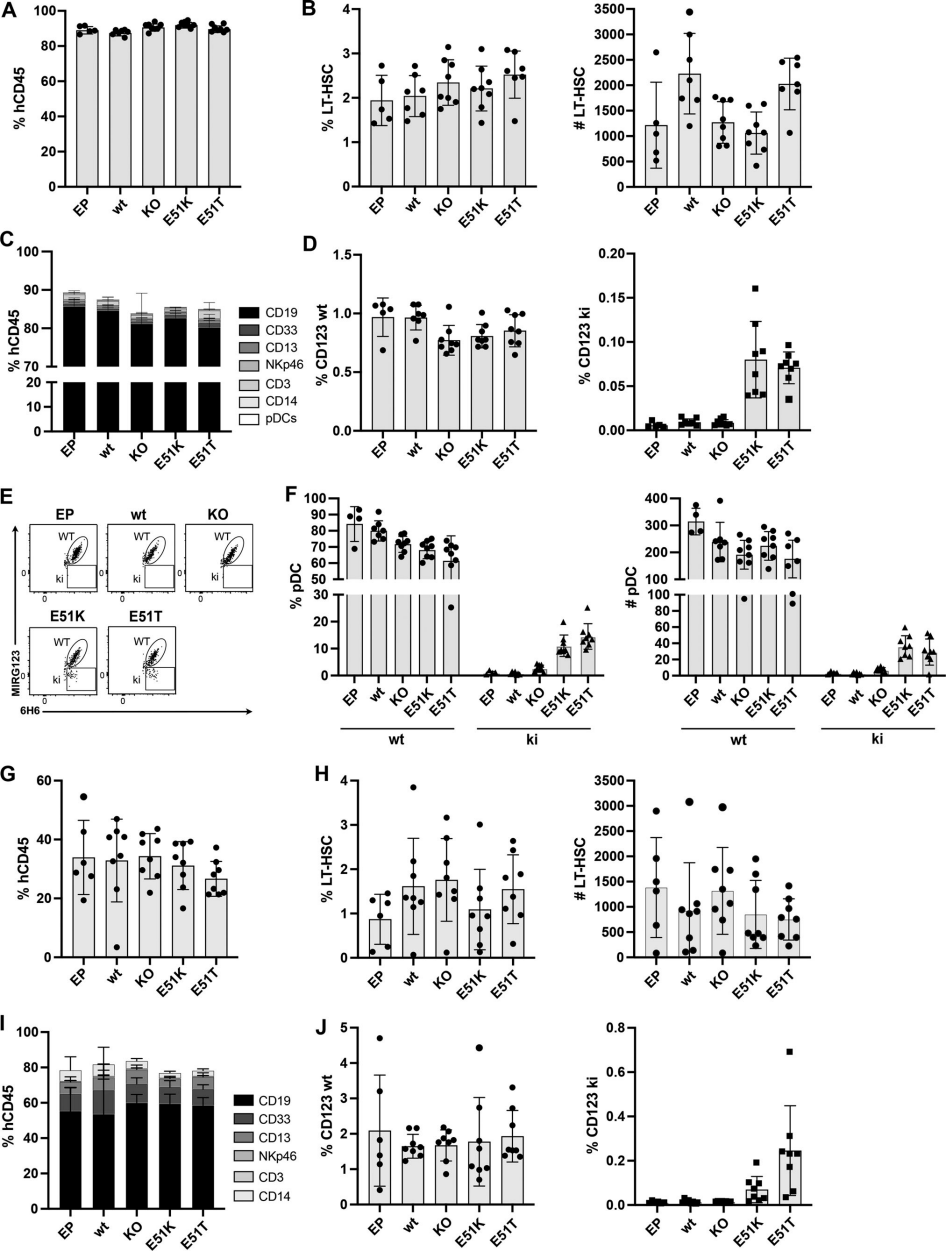
**CD123 epitope–engineered HSPCs engraft, differentiate normally, and possess long-term reconstitution potential in vivo. (A–F)** In vivo engraftment and differentiation potential of non-virally engineered HSPCs expressing E51K and E51T variants measured 16 wk after injection in NBSGW mice. **(A)** Human chimerism (% hCD45^+^) in bone marrow. **(B)** Proportion (left) and absolute number (right) of CD34^+^CD38^−^CD90^+^CD45RA^−^ HSCs in the bone marrow. **(C)** Multilineage differentiation in the spleen. Proportion of various differentiated cell subsets (CD19^+^, CD33^+^, CD13^+^, Nkp46^+^, CD3^+^, CD14^+^, pDCs) among human CD45^+^ cells are shown. **(D)** Proportion of wt CD123 (MIRG123^+^6H6^+^; left) and CD123 ki (MIRG123^−^6H6^+^; right) within human CD45^+^ cells in the bone marrow. **(E)** Representative dot plot of pDCs stained with MIRG123 and 6H6 in spleen. Gating strategy to identify pDCs depicted in Fig. S4. **(F)** Proportion (left) and absolute number (right) of pDCs. **(G–J)** Secondary transplant into NSG-SGM3 mice. Engraftment and differentiation were assessed 8 wk after the bone marrow transplant. **(G)** Human chimerism (% hCD45^+^) in bone marrow. **(H)** Relative fraction (left) and absolute number (right) of CD34^+^CD38^−^CD90^+^CD45RA^−^ HSCs in the bone marrow. **(I)** Multilineage differentiation in spleen. **(J)** Percentage of wt CD123 (MIRG123^+^6H6^+^; left) or edited CD123 ki (MIRG123^−^6H6^+^; right) among human CD45^+^ cells in bone marrow. **(A–J)** Error bars: mean (SD). **(A–F)** Data represent one out of three independent experiments, each performed with five to eight mice per group. **(G–J)** Data are from one experiment with five to eight mice per group.

**Figure S4.**
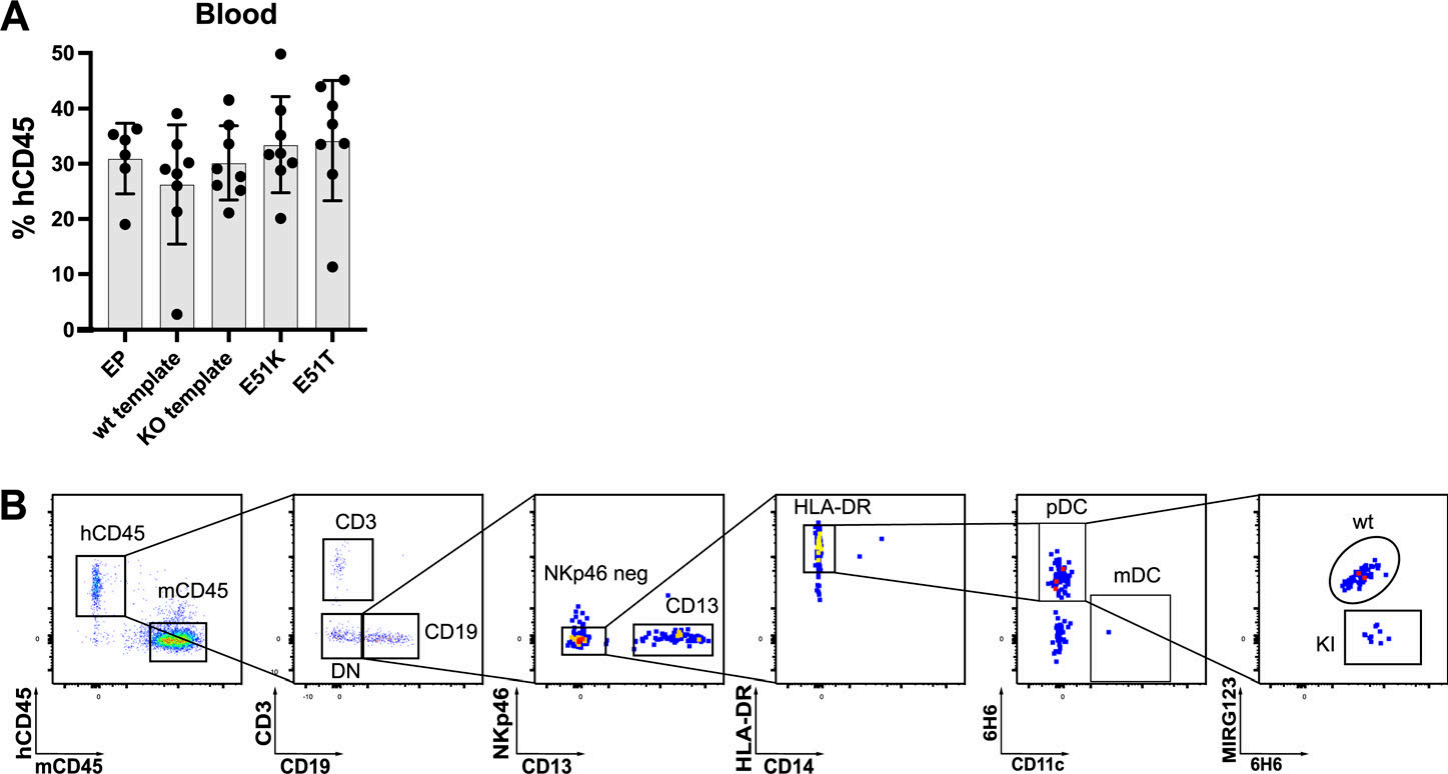
**Normal engraftment and differentiation of CD123-edited HSPCs. (A)** Human chimerism (% hCD45^+^) in blood. Data of a representative experiment performed with five to eight mice per group of three independent experiments. **(B)** Gating strategy to identify pDCs in the spleen 16 wk after injection of edited HSPCs in NBSGW mice. Error bars: mean (SD).

Next, we tested in vivo engraftment and differentiation potential. We injected the non-virally engineered HSPCs (EP, wt template, KO template, E51K, and E51T) into immunodeficient NBSGW mice. After 16 wk, all mice of the different groups showed comparable engraftment of hCD45^+^ cells in bone marrow (Fig. 6 A) and peripheral blood (Fig. S4 A). This was also reflected by a comparable frequency and number of human HSCs in the bone marrow (Fig. 6 B). Furthermore, all groups displayed multilineage differentiation in the spleen (Fig. 6 C). Next, we analyzed the presence of ki cells among hematopoietic cells in the bone marrow. hCD45^+^ cells expressing wt CD123 cells were found with comparable frequencies in all groups. In contrast, E51K and E51T were only found in mice that received ki HSPCs (Fig. 6 D). We then used the ability to discriminate ki cells from unedited cells to analyze cells with strong CD123 expression. Therefore, we analyzed CD123 on pDCs in the spleen (gating strategy, Fig. S4 B). Although pDC numbers were low, the typical MIRG123^−^6H6^+^ ki cells were only present in the mice injected with E51K or E51T HSPCs (Fig. 6, E and F). Thus, engineered HSPCs harboring single amino acid substitutions engrafted in vivo and differentiated into multiple cell types including pDCs.

To investigate whether LT-HSCs were correctly edited and functional, we performed secondary transplants. Secondary host mice (NSG-SGM3) transplanted with bone marrow cells from any of the five groups demonstrated comparable engraftment (Fig. 6 G) and frequency and numbers of LT-HSCs in bone marrow (Fig. 6 H). Furthermore, E51K- and E51T-engineered HSPCs gave rise to multilineage differentiation comparable with the wt template control mice in spleen (Fig. 6 I). Importantly, MIRG123^−^6H6^+^ ki cells were detectable in mice reconstituted with E51K and E51T HSPCs, respectively but not from any of the control HSPCs (Fig. 6 J). Thus, these data demonstrate that E51K and E51T were engineered into LT-HSCs that retained their engraftment potential and ki cells persisted long term.

### Engineered HSPCs enable tumor-selective CD123 immunotherapy

**Figure 7.**
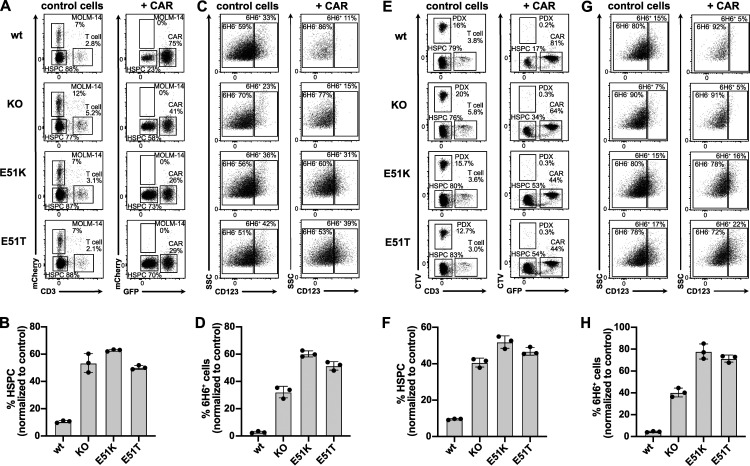
**Engineered HSPCs enable tumor-selective CD123 immunotherapy. (A–D)** Non-virally edited HSPCs co-cultured with MOLM-14-mCherry (AML cells) and control T cells (control cells) or 123CAR (+CAR) for 2 d. **(A)** Representative dot plots indicating proportion (%) of CD3^+^ T cells (control cells and CAR, respectively), MOLM-14 cells, and non-virally edited CD34^+^ HSPCs on day 2 of co-culture. **(B)** The proportion of HSPCs based on absolute counts from A normalized to the number of control T cells. **(C)** FACS plots illustrating the proportion (%) of wt or ki HSPCs at the end of the co-culture based on the binding characteristics to the mAb 6H6. Only clone 6H6 was used to avoid epitope masking by the 123CAR. **(D)** Fraction of 6H6^+^ cells based on absolute numbers from C relative to co-culture with control T cells. **(E–H)** Non-virally edited HSPCs co-cultured with PDX (CTV labeled) and control T cells or 123CAR for 2 d. **(E)** Representative dot plot indicating proportion (%) of CD3^+^ T cells, PDX-derived cells, and non-virally edited CD34^+^ HSPCs on day 2 of co-culture. **(F)** Quantification of HSPCs based on absolute counts from E. **(G)** Representative FACS data indicating the fraction (%) of wt or ki HSPCs based on the binding to the mAb clone 6H6 at the end of the co-culture. **(H)** Quantification of 6H6^+^ cells using absolute cell numbers from G relative to the co-culture with control T cells. **(A–H)** Error bars: mean (SD). **(A–D)** Data are from one out of two independent experiments, each performed in triplicate. **(E–H)** Data of one experiment performed in triplicate.

**Figure S5.**
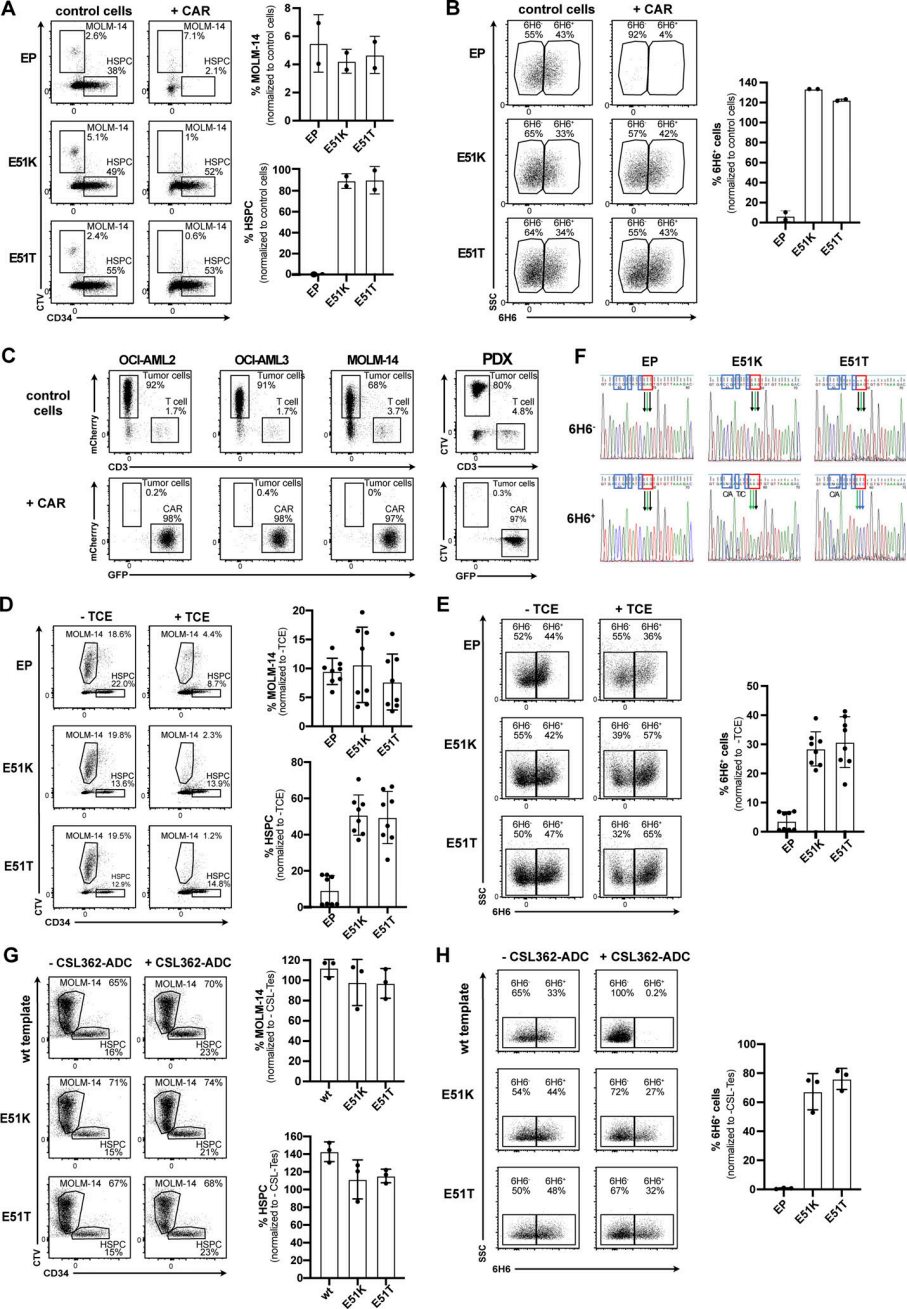
**Engineered HSPCs enable tumor-selective CD123 immunotherapy. (A and B)** Non-virally edited HSPCs co-cultured with MOLM-14 (CTV-labeled) and control T cells or 123CAR for 3 d. EP are electroporated but non-edited HSPCs. **(A)** Representative dot plots indicating MOLM-14 cells and non-virally edited CD34^+^ HSPCs on day 3 of co-culture (left) and the proportion thereof quantified using absolute cell numbers relative to control T cells (right). **(B)** FACS plots illustrating wt or ki HSPCs by their binding to the mAb 6H6 at the end of the co-culture (left), and proportion of 6H6^+^ cells based on absolute counts. **(A and B)** Data of one out of two independent experiments performed in triplicate. **(C)** 2-d co-culture of AML cells MOLM-14-mCherry, OCI-AML2-mCherry, OCI-AML3-mCherry, and PDX (CTV-labeled) with control T cells or 123CAR. Data of one experiment performed in triplicate. **(D and E)** Non-virally edited HSPCs co-cultured with MOLM-14 (CTV-labeled) and autologous T cells with or without CSL362/OKT3-TCE (100 ng/ml) for 3 d. Control conditions (EP) are electroporated but non-edited HSPCs. **(D)** Representative dot plot (left) and proportion (right) of MOLM14 cells and CD34^+^ HSPCs in different conditions on day 3 of co-culture with or without TCE. Representative data are from one out of three independent experiments with two different donors performed in triplicate. **(E)** FACS plot (left) and percentage (right) of edited HSPCs (6H6^+^ cells) at the end of the co-culture with or without TCE. One of two independent experiments, each performed in triplicate, are shown. **(F)** Sanger sequencing chromatogram of FACS-sorted 6H6^+^ and 6H6^−^ HSPCs on day 3 of co-culture with autologous T cells and CSL362/OKT3-TCE. Blue boxes: silent mutations; red boxes: E51K and E51T amino acid substitutions. Data are from one experiment. **(G and H)** Non-virally edited HSPCs co-cultured with MOLM-14 (CTV-labeled) with or without CSL362-ADC (10 nM) for 3 d. EP are electroporated but genetically not modified HSPCs. **(G)** Representative dot plot (left) and fraction (right) of MOLM14 cells and CD34^+^ HSPCs in different conditions on day 3 of co-culture with CSL362-ADC. Representative data are from one out of three independent experiments with two individual donors were performed in triplicate. **(H)** Flow cytometry data (left) and summary (right) showing the percentage of edited 6H6^+^ HSPCs at the end of the co-culture. Data are from one out of three individual experiments performed in triplicate. Error bars: mean (SD).

After establishing efficient engineering, a favorable safety profile and demonstration of preserved function and long-term engraftment potential of E51K and E51T HSPCs, we sought to investigate tumor selective CD123-targeted immunotherapy and resistance of engineered HSPCs. We co-cultured engineered HSPCs with control T cells not expressing a CAR (CD3^+^ CAR-negative) or 123CAR (GFP^+^) together with the AML cell line MOLM-14 (mCherry^+^). Tumor cells were cleared in all samples with 123CAR but not CAR-negative control T cells (Fig. 7 A). Quantification of HSPCs based on absolute cell counts revealed that wt template CD34^+^ HSPCs were depleted in comparison to KO HSPCs and E51K and E51T HSPCs (Fig. 7 B). Since HSPC KO/ki engineering usually remained <40% (Fig. 5, A–D), a twofold reduction of CD34^+^ HSPCs would be expected due to the killing of unedited, CD123 expressing HSPCs. To verify which cells were specifically eliminated, we compared the staining profile of CD123 on the remaining CD34^+^ HSPCs after co-culture. We stained CD123 using only mAb clone 6H6 to avoid possible epitope masking. HSPCs of all four groups incubated with CAR-negative control T cells expressed CD123 (Fig. 7 C). In contrast, 123CAR eliminated CD123 expressing cells in wt template HSPCs and KO template HSPCs but not E51K or E51T HSPCs. Thus, 123CAR preferentially depleted highly CD123 expressing HSPCs. However, quantification showed that wt template HSPCs were also depleted among the 6H6^−^ cells since very few cells remained (Fig. 7, C and D). In contrast, E51K and E51T HSPCs were resistant and continued to express CD123. In a similar experiment, we incubated MOLM-14 cells (CellTracker Violet, CTV) with non-engineered HSPCs (EP) and 123CAR cells in which case we observed near complete elimination of all EP control HSPCs while E51K and E51T HSPCs were resistant (Fig. S5, A and B). Together, these results suggest 123CAR-mediated bystander killing of HSPCs that express low or no CD123. 123CARs also efficiently depleted other AML cell lines (OCI-AML2, OCI-AML3) as well as patient-derived AML cells (designated PDX-derived cells; Fig. S5 C). Since PDX-derived cells were efficiently killed by 123CAR, we next mixed PDX-derived cells (CTV^+^) with edited HSPCs and 123CAR. As observed for MOLM-14, 123CARs indiscriminately killed PDX-derived cells as well as wt template HSPCs (Fig. 7, E and F). In contrast, 123CAR displayed selective cytotoxicity toward PDX-derived cells but preserved E51K and E51T HSPCs (Fig. 7, E–H).

Next, we investigated selective cancer immunotherapy by the TCE and ADC. The CSL362/OKT3-TCE was less potent than the 123CAR since some MOLM-14 cells remained (Fig. S5 D). Nevertheless, we also observed an overall depletion of EP HSPCs, suggesting some bystander killing of HSPCs expressing low or no CD123 (Fig. S5, D and E). To exclude possible artifacts due to epitope masking by remaining CSL362/OKT3-TCE that could block MIRG123 binding, we performed an experiment without MOLM-14 cells and FACS-sorted HSPCs based on 6H6 staining on the day of analysis. Sanger sequencing confirmed that EP HSPCs (6H6^−^ and 6H6^+^) and 6H6^−^ cells sorted from E51K and E51T HSPCs were primarily wt (Fig. S5 F). Thus, these are HSPCs that have a CD123 wt genotype but do not express CD123. In contrast, sorted 6H6^+^ ki HSPCs were almost purely E51K (AAG) or E51T (ACC), respectively. Thus, E51K and E51T expressing HSPCs withstood CD123-targeted TCE-mediated cytotoxicity leading to an enrichment of edited ki HSPCs. To test ADC as a depletion modality, we tested selective tumor killing with MOLM-14 and engineered HSPCs. The CD123-ADC depleted CD123-expressing 6H6^+^ HSPCs but not E51K or E51T HSPCs (Fig. S5 H). Thus, engineered ki HSPCs were also shielded from an ADC. However, MOLM-14 cells were not depleted and HSPC numbers for EP, E51K, and E51T remained the same (Fig. S5 G). Thus, we did not observe any bystander killing by this CD123-targeted ADC, but overall efficacy was limited. In summary, epitope-engineered, shielded HSPCs can safely be generated, are functional but resistant to CD123-targeted immunotherapy, and thereby enable tumor-selective targeting.

## Discussion

HSCT is a potentially curative approach for hematologic diseases. A conditioning phase prepares the transplantation of autologous or allogeneic HSCs which, after engraftment, will rebuild a new hematopoietic and immune system. The conditioning serves to remove host HSCs and can kill tumor cells when HSCT is applied to malignant diseases. However, current untargeted cytotoxic conditioning regimens have been directly or indirectly associated with transplant-related morbidity and mortality. With the advent of highly effective targeted depleting agents such as mAbs, ADCs, TCEs, and CAR T cells, it may become possible to replace untargeted conditioning and tumor control with antigen-specific immunotherapy (Czechowicz et al., 2007, 2019; Li et al., 2019; Myburgh et al., 2020; Palchaudhuri et al., 2016). However, the absence of suitable antigens constitutes a major impediment to progress in this field (Finck et al., 2022; Irving et al., 2022; Majzner and Mackall, 2019). In particular, targeting LSCs would be highly desirable to treat AML, but the risk for myelosuppression arising from shared antigen expression of LSCs and healthy HSCs precludes continuous posttransplant therapy (Myburgh et al., 2020). As a compromise, it was proposed to use CAR T cells directed against CD123 or CD117 to purge tumor cells and HSCs alike, followed by myeloablative conditioning to remove the CAR T cells before HSCT (Arai et al., 2018; Gill et al., 2014; Myburgh et al., 2020). Recently, CAR T cells targeting CD7 were used clinically to deeply purge relapsed T cell acute lymphoblastic leukemia (Chiesa et al., 2023). However, the CAR7 was only used for a limited time before HSCT and they led to multilineage cytopenia. Thus, the targeted immunotherapy was limited to the pretransplant period and only served as a bridge-to-transplant. However, the ability to continue immunotherapy posttransplant would be highly desirable to eliminate minimal residual disease and prevent relapse long-term.

We previously used genome engineering to substitute single amino acids in surface proteins of murine T cells which completely abolished binding of specific mAbs (Kornete et al., 2018). Since we converted protein variants known to have an exchangeable function (also known as congenic markers), the results suggested that engineering a minimal alteration in surface proteins could enable safe cell shielding for therapeutic purposes. To enable HSCT with continuous antigen-specific immunotherapy, we engineered human HSPCs to express epitope-engineered CD123 and demonstrate that these cells are both shielded and functional, respectively. We used structural information to identify at least one amino acid substitution that completely abrogated mAb binding for three separate residues. This is important since it increases the chances that an appropriate genome engineering strategy can be found to safely and efficiently insert a variant into primary therapeutic cells. The choice of the most suitable genome engineering approach will depend on the desired amino acid substitution and cell type. To establish feasibility, we employed CRISPR/Cas9-mediated HDR, which provides flexibility and precise programmability through a DNA template encoding the desired amino acid exchange. Despite some shortcomings such as cellular toxicity, HDR-mediated HSPC editing is safe and clinically relevant (Cromer et al., 2022; Dever et al., 2016). In fact, we did not find any detectable off-target activity using multiple complementary assays, demonstrating the safe engineering of HSPCs. In addition, more recently developed genome engineering approaches provide additional options. Specifically, base editing is well tolerated, can achieve high editing efficiencies in HSPCs, and is suitable for multiplexed editing (Anzalone et al., 2020). However, base editors have different constraints than HDR since they typically only convert A→G or C→T and also have PAM restrictions, and the editing window—despite being limited—can result in unwanted bystander editing. As a consequence, base editing can only install a fraction of desired codon changes for a particular amino acid change. Indeed, we found that these inherent limitations are relevant when using base editors to install cell shielding variants because the most suitable amino acid substitutions may not be accessible to base editing or bystander editing may affect the function of the engineered protein (unpublished data). Therefore, careful characterization of the editing outcome and the resulting protein variants is required (unpublished data). Prime editing employs a templated approach that also avoids dsDNA breaks and provides the greatest flexibility to install any desired codon change and could therefore be suitable for epitope engineering (Anzalone et al., 2020). However, prime editing was only recently shown to work in HPSCs, and further investigation is needed (Everette et al., 2023).

Information about naturally occurring polymorphisms without known disease association (e.g., CD123 E51K) could inform variant choice from a safety perspective. For instance, although IL-3 binding to CD123 E51K may be slightly reduced compared with wt CD123 (Fig. 4 D), it appears that different binding strengths to IL-3 may be biologically tolerated. Theoretically, allogeneic HSPC donors could be prescreened for such variants, which would provide shielding without the need to engineer the cells. However, practically, most polymorphisms are likely too rare since HSPCs also have to be matched for HLA. Conversely, this indicates that some patients receiving therapeutic cell-depleting agents may be non-responders due to genetic variation. Remarkably, our results show that a single amino acid substitution is sufficient to protect from mAb (ADCC), ADC, TCE, and even CAR T cells. Thus, shielded HSPCs or other therapeutic cells could be combined with a broad range of available cell-depleting agents. However, our data shows that careful characterization of the variants for potential clinical translation is important. For instance, although R84E was shielded from all tested depletion modalities, it showed signs of reduced stability. Furthermore, S59P and R84E both showed reduced IL-3 binding and therefore were not further pursued. In addition, the degree of binding reduction will be relevant for the choice of the mode of action of the depleter. For instance, S59Y, R84T, and R84Q were protected from ADCC and TCE but led to substantial killing by the CAR T cells. It is noteworthy that the CAR T response was rather digital with non-binders being protected whereas weak binders resulted in strong CAR T activation and cytotoxicity. In contrast, responses to weak binders were more analogous for ADCC and TCE, resulting in much more limited killing. The sensitivity of CAR T responses even to weak binding will also be important when considering targets with expression by non-hematopoietic cells. For instance, CD123 expression in endothelial cells could result in toxicity that cannot be addressed by the approach presented here since endothelial cells are non-hematopoietic and will therefore not be resistant. Another example is fms-like receptor tyrosine kinase (Flt3), which could be an attractive target to treat AML. However, inhibitors may lead to acute cardiotoxicity (Kim et al., 2021). It should be noted that Flt3 expression was found in murine cardiomyocytes, and expression in human induced pluripotent stem cell–derived cardiomyocytes was sufficient to result in mild but detectable activation of Flt3-targeted CAR T cells (Niswander et al., 2023; Pfister et al., 2014). Therefore, target expression in non-hematopoietic cells potentially constitutes a significant limitation that needs to be carefully assessed when evaluating target antigens and bears the risk of precluding long-term therapy. Thus, the therapeutic window created by a shielding variant will be a function of the binding reduction between the wt and the engineered variant, the expression on the target cells (which may be dynamic), expression on non-hematopoietic cells, as well as the efficacy of the depleting agent. In some cases, e.g., when a blocking mAb is used, residual binding of the shielding variant may be acceptable. In others, when the variant is to be paired with a highly effective depleter (e.g., ADC or CAR T), the non-binding must be more stringent.

To fully exploit the advantage of combining shielded HSPCs with targeted immunotherapy, the function of the antigen should be preserved (Finck et al., 2022). Our results show that CD123-deficient HSPCs had a competitive disadvantage in vitro and CD123 KO HSPCs were strongly depleted. In contrast, selected shielded CD123 single amino acid substitution variants preserved IL-3 binding, signaling (p-STAT5), and IL-3 dose-dependent growth. Furthermore, engineered ki HSPCs differentiated normally in vitro, engrafted, and displayed multilineage differentiation potential in vivo. Secondary transplantation experiments confirmed the successful engineering of long-term reconstituting LT-HSCs. However, it is important to note that additional studies will be needed for clinical translation. The xenotransplantation models used here have clear limitations and do not allow a definitive conclusion as to whether the engineered HSPCs are fully functional in the long run. The shielding variants may be immunogenic but since host mice are immunodeficient, xenotransplantation cannot be used to address immunogenicity. However, immunologic rejection is likely not a major concern since (i) immune cells with defined minor mismatches (congenic markers) are regularly transplanted to MHC-matched immunocompetent host mice without signs of rejection, (ii) transplanted HSC will likely induce tolerance to the variant, and (iii) in an allogeneic setting, mismatched HLA will be highly immunogenic requiring appropriate immunosuppression. Non-human primate models should be considered but may not be suitable since epitope engineering is focused on non-conserved regions. Tumorigenicity studies should be performed but ultimately clinical trials are needed. If safe, molecularly shielded HSPCs could allow tumor-selective CD123 targeted immunotherapy and in parallel enable rebuilding a CD123 variant–expressing hematopoietic system. For instance, CD123 targeting therapies are clinically successful for the treatment of BPDCN, a pDC-derived neoplasm. However, healthy pDCs strongly express CD123 and therefore will be depleted by any effective CD123 immunotherapy. Yet, pDCs are primary producers of type I IFN and hence anti-viral immunity. Their importance was recently illustrated for the protective immunity against SARS-CoV-2 (Asano et al., 2021). Therefore, transplanting CD123-shielded HSPCs may in the future potentially allow to fully restore immunity with a completely functional hematopoietic system while simultaneously allowing efficient tumor immunotherapy. More broadly, we envision that function-preserving but shielding variants could be identified for other proteins that currently cannot safely be targeted, e.g., CD45 or CD117. Applications could include T cell malignancies since T cell deficiency results in unacceptably severe immunosuppression and T cell function cannot readily be replaced. CD7 is of interest to treat T cell malignancies and AML, but CAR7 is currently only used as a bridge-to-transplant (Chiesa et al., 2023). Importantly, preserving the function of the engineered proteins could enable multiplex shielded HSPCs that are resistant to combination immunotherapies to address disease heterogeneity. However, the safety of multiplex-edited HSPCs would need to be assessed carefully. Finally, epitope shielding could open up the development of new immunotherapies against as yet unidentified targets and may be applicable for the replacement of other cell types e.g., T, B, and the many other immune cells that are currently being considered for engineered cellular therapies (Finck et al., 2022).

## Materials and methods

All sequences of HDRT, gRNAs, and oligos are listed in Table S4 A, and all flow cytometry antibodies used in the study are indicated in Table S4 B.

### Structural dataset and computational design of CD123 protein variants

Experimentally determined three-dimensional structures of the CD123 protein were retrieved from the PDB and include (i) the CD123-CSL362 complex in open and closed conformation (PDB ID: 4JZJ; Broughton et al., 2014), (ii) the CD123-IL-3 binary complex (PDB ID: 5UV8; Broughton et al., 2018), and (iii) the CD123 unbound structure extracted from the complex in the open and closed conformations. Given the structure of the complexes, the CSL362 and IL-3 epitopes were defined as the sets of CD123 residues having at least one atom within 4 Å of any CSL362 and IL-3 atoms, respectively. Per-residue relative solvent accessibility area (RSA) was computed using the Lee & Richards algorithm (Lee and Richards, 1971) implemented in FreeSASA (Mitternacht, 2016) using default parameters and upon removing crystallographic waters, sugars, and ions. Comprehensive mutagenesis was performed in silico using the EVmutation sequence-specific probabilistic model (Hopf et al., 2017) and the mutational effect, defined as ΔE, estimated using the epistatic coevolutionary analysis framework (Dunn et al., 2008; Ekeberg et al., 2013; Hopf et al., 2019). For a given mutant, the ΔE is computed as the sum of differences of the constraints on individual amino acid sites plus the sum of differences of the coupling parameters computed for all pairs of sites involving the mutated site. To quantify the total epistatic constraint acting on a given amino acid site of interest, evolutionary coupling analysis was run on a multiple sequence alignment spanning the entire CD123 sequence. The multiple sequence alignment was built using five iterations of the jackhammer HMM search algorithm against the non-redundant UniProtKB database (Suzek et al., 2015) and the default significance score for the inclusion of homologous sequences. As values of ΔE below, equal, and above 0 correspond to putatively beneficial, neutral, and deleterious effects, respectively, variants at a given site were selected upon ranking for decreasing ΔE.

### Eukaryotic cell lines

Freestyle CHO-S cells were purchased from Thermo Fisher Scientific (Cat#R80007) and were expanded in PowerCHO 2 Serum-free Medium (BELN12-771Q; Lonza) supplemented with GlutaMAX (Gibco), HT supplement (Cat# 41065012), and antibiotic–antimycotic (Cat#15240062) to a maximum density of 20 × 10^6^ cells/ml. TF-1 was purchased from DSMZ (Cat#ACC334) and maintained in RPMI-1640 media (Sigma-Aldrich) supplemented with 10% heat-inactivated FCS (Gibco Life Technologies), 2 mM GlutaMAX (Gibco Life Technologies), and 2 ng/ml hGM-CSF (215-GM; Bio-Techne). HEK-293 cells were a kind gift from M. Zavolan (Biozentrum Basel, Basel, Switzerland) and cultured in Dulbecco’s Modified Eagle’s Medium; high glucose (Sigma-Aldrich), supplemented with 10% heat-inactivated FCS, and 2 mM GlutaMAX. MOLM-14 were purchased from DSMZ (Cat#ACC777), and K562 wase purchased from ATCC (Cat#CCL243). Both cell lines were maintained in RPMI-1640 media supplemented with 10% heat-inactivated FCS and 2 mM GlutaMAX. All cell lines were freshly thawed and passaged three to six times prior to use. AML cell lines (MOLM-14, OCI-AML-2, and OCI-AML-3) were retrovirally transduced with MI-Luciferase-IRES-mCherry (gift from Xiaoping Sun, MD Anderson Cancer Center, Houston, TX, USA; plasmid #75020; Addgene; http://n2t.net/addgene:75020; RRID:Addgene_75020; Mallampati et al., 2014). Cells were then FACS-sorted based on mCherry expression. After expansion, cells were short tandem repeat profiled and tested mycoplasma negative before being frozen until further use.

### Hematopoietic cells from human subjects (PDX-derived cells)

Deidentified patient-derived AML samples were obtained from the PDX repository (Cancer Research Center of Toulouse, France; Larrue et al., 2023; Sabatier et al., 2023). Cells were propagated in NSG mice for two generations. Therefore, we refer to these cells as PDX-derived since they are patient-derived AML cells that were xenografted to mice. A signed written informed consent for research use in accordance with the Declaration of Helsinki was obtained from patients and approved by the Geneva Health Department Ethic Committee.

### Cloning and expression of recombinant wt human CD123 and its variants

Full-length cDNA of human CD123 (NM_002183.2) was obtained from a pCMV3 vector (Cat#HG10518-M; Sino Biological). The hygromycin sequence was replaced by a neomycin resistance cassette by Gibson Assembly. The point mutations of the human CD123 variants were introduced into the vector using PCR (see Table S4 A)*.* 2 × 10^6^ HEK-293 cells were electroporated with the pCMV3 vector encoding CD123 wt or its variants using the Neon Transfection System (1,100 V, 20 ms, 2 pulses; Thermo Fisher Scientific). To generate stable cell lines, Geneticin G418 (50 mg/ml; BioConcept) was added to the cell culture medium at a concentration of 350 µg/ml.

### Cloning and expression of the mAb CSL362 biosimilar (MIRG123)

The heavy and kappa light chain variable regions (VH and VKL) of CSL362 were derived from the CSL362/OKT3-TCE (Hutmacher et al., 2019). To generate the monoclonal IgG1 antibody CSL362 biosimilar MIRG123, the VH and VKL sequences were cloned into AbVec2.0-IGHG1 (was a gift from Hedda Wardemann, Deutsches Krebsforschungszentrum Heidelberg, Heidelberg, Germany; plasmid #80795; Addgene, http://n2t.net/addgene:80795; RRID:Addgene_80795) and AbVec1.1-IGKC (was a gift from Hedda Wardemann, plasmid #80796; Addgene, http://n2t.net/addgene:80796; RRID:Addgene_80796), respectively (Tiller et al., 2008). 2 × 10^9^ Freestyle CHO-S cells were resuspended in 500 ml ProCHO 4 Protein-free Medium (Cat#BEBP12-029; Lonza) supplemented with 1XHT, GlutaMAX, antibiotic–antimycotic and co-transfected with both plasmids (0.6 mg each) using 5 mg polyethyleneimine (PEI, Cat#23966; Polysciences). The cells were expanded under constant rolling (140 rpm) at 31°C, 5% CO_2_ for 6 d. The Freestyle CHO-S cells were pelleted, and the filtered supernatant (0.22 μm filter) was applied to a Protein A column for purification. CSL362 biosimilar was eluted with 0.1 M glycine (pH = 2.2), 0.5 ml fractions were collected, and OD_280_ was measured using a Nanodrop spectrophotometer. The high-concentration fractions were pooled and dialyzed twice overnight in PBS.

### Expression and purification of CSL362/OKT3-TCE

The design of the CSL362/OKT3-TCE was recently described (Hutmacher et al., 2019) and the plasmid was a kind gift from D. Neri (ETH Zurich, Zurich, Switzerland). Freestyle CHO-S producer cells were transfected using PEI (as described above) with 1.7 mg TCE DNA. Following 6 d of expansion, the filtered cell culture supernatant was applied to a 5 ml Ni-NTA column (Thermo Fisher Scientific) prewashed with 100 ml washing solution (PBS, 150 mM NaCl, 5 mM imidazole, pH7.4) for protein purification. The eluted and dialyzed TCE was filtered (0.22 μm) and stored in aliquots (1 mg/ml) at −80°C.

### Flow cytometry and cell sorting

Flow cytometry was performed on BD LSRFortessa with the BD FACSDiva Software and the data were analyzed with FlowJo Software. Antibodies used for flow cytometry can be found in the Table S4 B. For cell sorting, the cells were pelleted and resuspended in FACS Buffer (PBS + 2% FCS) supplemented with 1 mM EDTA, and sorting was performed either on BD FACSAria or BD FACSMelody Cell Sorter. The control cells were also subjected to the sorting process.

### Primary human T cell isolation and culture

Leucocyte buffy coats from anonymous healthy human donors were purchased from the blood donation center Basel (Blutspendezentrum SRK beider Basel, BSZ). Peripheral blood mononuclear cells (PBMCs) were isolated by density centrifugation using SepMate tubes (StemCell Technologies) and the density gradient medium Ficoll-Paque (GE Healthcare) according to the manufacturer’s protocol. Human T cells were purified (>96% purity) by magnetic negative selection using an EasySep Human T Cell Isolation Kit (Cat#17951; StemCell Technologies) according to the manufacturer’s instructions. If frozen PBMCs were used, T cells were isolated after thawing and cultured in supplemented media without stimulation overnight. T cells were cultured in RPMI-1640 Medium (Sigma-Aldrich) supplemented with 10% heat-inactivated human serum (AB^+^, male; purchased from BSZ Basel), 10 mM HEPES (Sigma-Aldrich), 2 mM GlutaMAX, 1 mM sodium pyruvate, 0.05 mM 2-mercaptoethanol, 1% MEM non-essential amino acids (100×; all Gibco Life Technologies), and IL-2 150 U/ml (proleukin; University Hospital Basel). The medium and IL-2 were replenished every 2 d, and the cells were kept at a cell density of 1 × 10^6^ cells/ml.

### ADCC assay (FcγRIIIa activation assay)

ADCC assay was performed using the ADCC Reporter Bioassays, V Variant (Cat#G7015; Promega) according to the manufacturer’s instructions. Target cells HEK-293 expressing the CD123 variants were seeded in a white 96-well plate clear bottom at 4,400 cells/100 μl culture medium. On day 1, the medium was removed and effector cells (Jurkat/FcγRIIIa/NFAT-Luc cells; effector-to-target ratio 12:1) and MIRG123 antibody (final concentration 1 μg/ml) were added and incubated for 5 h at 37°C 5% CO_2_. The emitted luminescence was read 10 min after the addition of Bio-Glo Luciferase Assay Reagent (Promega) using the PHERAstart FSX (BMG LABTECH) program Luc-Glo (LUM), GainA = 3,600, optic module = LUMplus. Raji cells incubated with 1 μg/ml of anti-CD20 antibody rituximab were used as positive control.

### In vitro TCE-mediated killing assay with primary T cells

For the TCE killing assays the HEK-293 target cells were co-cultured together with human effector T cells and the CD3/CSL362-TCE at an effector-to-target ratio of 10:1 for 72 h. 1d prior to co-culture HEK, HEK-CD12,3 and the HEK expressing the CD123 variants were stained with CTV according to the manufacturer’s protocol. The following day effector T cells were added with the TCE at a concentration of 300 ng/ml and kept for 72 h at 37°C. Cytotoxic activity and activation of T cells were analyzed by flow cytometry. The specific killing was calculated as follows: (1 − no. alive target cells with TCE/no. alive target cells without TCE) × 100. Cell morphology was assessed with the light microscope Axio Vert.A1 (Zeiss) at 20× magnification.

### Design and production of the CD123CAR HDRT

The CAR T cells were generated by co-EP of CRISPR-Cas9 ribonucleoproteins (RNPs) specific for the *TRAC* locus and a double-stranded DNA HDR template (HDRT). The HDRT encodes a second-generation CD123-specific CAR with the scFv of clone CSL362, the CD8α hinge and transmembrane domain (Gen CD8A ENSG00000153563), the intracellular signaling moieties 4-1BB (Gen TNFRSF9 ENSG00000049249), and CD3ζ (Gen CD247 ENSG00000198821), as well as the fluorescent reporter protein GFP (for full sequence, see Table S4 A). It is flanked by symmetric arms of homology (300 bp) complementary to the *TRAC* locus Exon 1. The construct was synthesized by GenScript and the plasmid was used as a template for PCR amplification (Kapa Hifi Hotstart Ready Mix, Cat#F-530-L; Roche). The PCR amplicon was purified with NucleoSpin Gel and PCR clean-up kit (Macherey-Nagel) according to the manufacturer’s instruction and the correct size was verified by gel. The HDRT was then condensed to a final concentration of 1 µg/µl using vacuum concentration and stored at −20°C until usage.

### Engineering primary CD123CAR T cells

Protocols for human CRISPR/Cas9-mediated genome engineering are based on Roth et al. (2018, 2020). In short, Cas9 RNPs were freshly generated prior to each EP. Thawed crRNA and tracrRNA (purchased from IDT Technologies; at 200 µM) were mixed in a 1:1 M ratio (120 pmol each), denatured at 95°C for 5 min, and annealed at room temperature (RT) for 10–20 min to complex an 80-µM gRNA solution. Polyglutamic acid (15–50 kD at 100 mg/ml; Sigma-Aldrich) was added to the gRNA in a 0.8:1 volume ratio (Nguyen et al., 2020). To complex RNPs, 60 pmol recombinant Cas9 (University of California Berkeley; at 40 µM) was mixed with the gRNA (molar ratio Cas9:gRNA = 1:2) and incubated for 20 min at RT. Prior to EP, isolated human T cells were activated for 48 h with CD3/CD28 Dynabeads (Thermo Fisher Scientific) at a cell to beads ratio 1:1 together with the recombinant human cytokines IL-2 (150 U/ml), IL-7 (5 ng/ml; R&D Systems), and IL-15 (5 ng/ml; R&D Systems). Electroporation was performed with the 4D-Nucleofector system (Lonza) with Program EH-115. Following activation, the T cells were debeaded using an EasySep magnet, and 1 × 10^6^ cells were resuspended in 20 µl Lonza-supplemented P3 EP buffer. HDRT (3–4 µg) and RNPs (60 pmol) were mixed separately and incubated for 5 min. The cells were added to the mix and the total volume was transferred to 16-well Nucleocuvette Strips. Immediately following EP, 80 µl of prewarmed supplemented medium was added to each cuvette and incubated at 37°C. After 20 min, the cells were transferred into 48-well culture plates and replenished with IL-2 500 U/ml. Following flow sorting at day 3–5 after EP, the cells were expanded for 5–6 d until used for subsequent experiments. Control T cells were electroporated with an incomplete RNP (missing the specific crRNA), otherwise processed as the CAR T cells.

### In vitro human CD123CAR killing assay

The day before co-culture, HEK-293 target cells were stained with CTV and kept in a supplemented human medium overnight. The flow-sorted, expanded GFP^+^ CAR T cells and control cells were added to the target cells in an effector-to-target ratio 10:1 and co-cultured for 24 h. Specific killing and T cell activation were measured by flow cytometry. Specific killing was calculated according to the indicated formula: (1 − no. alive target cells in co-culture with CAR T cells/no. alive target cells in co-culture with control cells) × 100. Using the microscope Axio Vert.A1 (Zeiss), cell morphology was recorded.

### In vitro 123CAR-mediated killing assay of non-virally engineered HSPCs

For the 123CAR killing assays, the HSPCs were edited as described below. 2 d after EP edited HSPCs, MOLM-14-mCherry, OCI-AML2-mCherry, OCI-AML3-mCherry, or PDX-derived cells (labeled with CTV according to the manufacturer’s instruction) were co-cultured with control T cells or 123CAR in a 96 U-bottom plate at an effector to target ratio of 3:1 for 48 h at 37°C. In a set of experiments, MOLM-14-mCherry or PDX-derived cells (labeled with CTV according to the manufacturer’s instruction) were co-cultured with edited HSPCs and T cells or 123CAR cells at an effector to target to tumor ratio of 3:0.5:0.5 together. Cytotoxic activity (specific killing and elimination of non-edited HSCs) was analyzed by flow cytometry.

### Human cytokine measurement (ELISA)

IFNγ was measured from the supernatants of the co-culture experiments (TCE/CAR) using the colorimetric ELISA MAX Standard Set Human IFNγ kit (BioLegend) according to the manufacturer’s instructions. The optical density was read at 450 nm with the microplate reader. A standard curve calculated from standard dilutions was run in duplicates with every experiment.

### Genomic DNA extraction and sequencing from human T cells, eukaryotic cell lines, and HSPCs

Genomic DNA was extracted using the QuickExtract (QE09050; Biosearch technologies) according to the manufacturer’s instructions. Alternatively, cells were lysed in Tail Lysis Buffer (100 mM Tris [pH 8.5], 5 mM Na-EDTA, 0.2% SDS, 200 mM NaCl) containing Proteinase K (0.1 µg; Sigma-Aldrich) at 56°C (1,000 rpm). The DNA was precipitated with isopropanol (1:1 volume ratio) and washed in 70% ethanol. The genomic DNA concentration was measured with a NanoDrop device (Thermo Fisher Scientific). PCR was performed using either GoTaq G2 Green Master Mix (Cat#M782B; Promega) or Kapa Hifi Hotstart Ready Mix. Sanger Sequencing was performed at Microsynth AG Switzerland. Sequences were analyzed using MegAlign Pro (DNASTAR).

### NGS

Genomic DNA was isolated from HSPCs using Quick extraction buffer. The targeted amplicons library was prepared following Illumina’s recommendation using a two-step PCR protocol. Briefly, nested PCRs were performed on each DNA sample using the HiFi KAPA polymerase (Roche). Following Illumina barcoding (Nextera indices; Illumina), PCR samples were pooled, beads were purified and quantified using Qubit dsDNA BR (Thermo Fisher Scientific). The library was sequenced on an Illumina MiSeq instrument using Illumina MiSeq Reagent Kit v2 Micro (300 cycles) with 50% PhiX spike-in (Illumina). After demultiplexing, each sample was assessed for quality and analyzed using CRISPResso v2 (Clement et al., 2019). For each of the samples, we provided the HDRT, the reference sequence, and the guide sequence, and applied a minimum base quality of Phred 25. We used a custom R script to quantify each allele within a quantification window of four nucleotides including one silent mutation followed by the targeted amino acid.

### BLI measurement of binding

All BLI measurements were performed either on an Octet Red96e (ForteBio) or on an Octet R8 (Sartorius). The ECD of CD123 wt and variants were produced and purified by Icosagen.

### CSL362 hIgG1 binding to CD123 wt ECD and variants

Binding of antibody CSL362 hIgG1 to CD123 wt ECD and variants (analytes in solution) was performed at low (50 nM) and high (300 nM) concentrations of the analyte. Antibody CSL362 hIgG1 (captured ligand) was captured by Anti-Human Fc capture biosensor (AHC; Sartorius, PN: 18-5060) for 300 s at 0.5 µg/ml. Analytes CD123 wt ECD and variants were titrated at seven concentrations (1:2 dilution series) from 50 to 0.78 nM and from 300 to 4.7 nM. Association to analyte was monitored for 300 s and dissociation for 600 or 900 s. Double reference subtraction was performed against buffer only and the biosensor was loaded with a negative hIgG1 control. Regeneration was performed in 10 mM Gly-HCl pH1.7. Data were analyzed using the Octet Data Analysis software HT 12.0. Data were fitted to a 1:1 binding model. Kinetic rates k_a_ and k_d_ were globally fitted.

### 6H6 mIgG1 binding to CD123 wt ECD and variants

Binding of antibody 6H6 mIgG1 (PN: 306002; captured ligand; BioLegend) to CD123 wt ECD and variants (analytes in solution) was performed using Streptavidin capture biosensor (SA; Sartorius, PN: 18-5019). CaptureSelect Biotin Anti-LC-kappa (Murine; PN: 7103152100; Thermo Fisher Scientific) was captured for 600 s at 1 µg/ml on SA tips. Those biosensors were then used to capture antibody 6H6 mIgG1 for 300 s at 2.5 µg/ml. Analytes CD123 wt ECD and variants were titrated at seven concentrations from 50 to 1.56 nM. Association to analyte was monitored for 300 s and dissociation for 600 s. Buffer-only well was used as a reference. Regeneration was performed in 10 mM Gly-HCl pH1.7. Data were analyzed using the Octet Data Analysis software HT 12.0. Data were fitted (when possible) to a 1:1 binding model. Kinetic rates k_a_ and k_d_ were globally fitted.

### IL-3 binding to CD123 wt ECD and variants

Binding of IL-3 (PN: 11858-H08H; Sino Biological; analyte in solution) to CD123 wt ECD and variants (captured ligands) was performed using Streptavidin capture biosensor (SA; Sartorius, PN: 18-5019). CD123 wt ECD and variants were biotinylated using Biotinylation kit Type B (PN: ab201796; Abcam) following manufacturer instructions. Biotinylated CD123 wt ECD and variants (ligands) were captured on SA tips for 1,000 s at 3 µg/ml concentration. Analyte IL-3 was titrated at seven concentrations from 500 to 7.8 nM in PBS pH7.4. Association to analyte was monitored for 300 s and dissociation for 120 s. Buffer-only well was used as a reference. No regeneration was performed, and a new set of tips was used for each biotinylated captured ligand. Data were analyzed using the Octet Data Analysis software HT12.0. Due to the fast on/off nature of the interaction, data were analyzed using steady-state analysis.

### Thermal stability analysis

DSF analysis was performed on a Bio-Rad CFX96 Touch Deep Well RT PCR Detection System. Sypro Orange 5,000× in DMSO (PN: S5692; Sigma-Aldrich) was used at a final concentration of 5×. The temperature gradient was performed from 25°C to 95°C in increments of 1.5°C in a reaction volume of 20 μl. “FRET” scan mode was used to monitor fluorescence. All samples were analyzed at a final concentration of 0.25 mg/ml in triplicate in PBS pH7.4. The temperature of protein unfolding transition (Tm) was calculated using the first derivative method.

### Engineering of TF-1 cells expressing CD123 variants and functional assays thereof

RNPs were freshly prepared as outlined above. 50 pmol ssDNA HDRT (180 bp length, Ultramer DNA Oligonucleotides, synthesized by IDT) were added to the RNPs. Per reaction, 0.2 × 10^6^ TF-1 cells were resuspended in 10 µl R buffer (Neon Transfection System) and electroporated using the Neon Transfection System (1,200 V, 40 ms, 1 pulse). Following an expansion period of 12 d, the edited cells were flow-sorted based on the binding to MIRG123 and 6H6: wt (MIRG123^+^6H6^+^), KO (MIRG123^−^6H6^−^), and ki (MIRG123^−^6H6^+^).

To test the responsiveness of TF-1 cells to human IL-3, 0.18 × 10^5^ sorted cells were distributed into white 96-well plate clear bottom tissue-culture treated (Greiner), and different IL-3 concentrations indicated in Fig. 5 B were added. In certain experiments, MIRG123 was added in the concentrations shown in Fig. 5 C. After 3 d, proliferation was assessed using CellTiter-Glo 2.0 (Cat#G9241; Promega) according to the manufacturer’s protocol. Luminescence was assessed with the Synergy H1 (BioTek) with an integration time of 1 s.

### Non-virally CRISPR/Cas9 mediated engineering of HSPCs

Leukopaks were purchased from CytoCare and HSPCs were isolated by the LP-34 Process using the CliniMACS Prodigy (Miltenyi). HSPCs were thawed in HSC-Brew GMP Basal Medium (Miltenyi) supplemented with HSC-Brew GMP Supplement, 2% human serum albumin, 100 ng/ml stem cell factor (SCF), 100 ng/ml thrombopoietin (TPO), 100 ng/ml Fms-like tyrosine kinase 3 ligand (Flt3L), and 60 ng/ml IL-3 (Miltenyi) at a concentration of 0.5 × 10^6^ cells/ml. Cells were electroporated 2 d later. gRNAs were freshly prepared as outlined above, but 50 µM crRNA and tracrRNA were used to form the gRNA and complexed with 1 µM Spyfi Cas9 (Aldevron at 61.889 µM) at a molar ratio Cas9:gRNA = 1:2 and incubated for 20 min at RT. As a control, incomplete RNPs lacking the site-specific crRNA were generated. During the RNPs complexing, HSPCs were collected, washed twice with EP buffer (Miltenyi), and resuspended in EP buffer at 1 × 10^6^ cells/90 µl. Cells were then mixed with 5 µl RNP and ssDNA HDRT encoding the variants (5 µl corresponding to 500 pmol), and the whole volume was transferred into the EP nucleocuvette. Electroporation was performed with the CliniMACS Prodigy (600 V 100 μs burst/400 V 750 μs square). Immediately after EP, the cells were transferred to a six-well plate and rested for 20 min at RT. After 20 min, 2 ml of prewarmed HSC medium supplemented with 100 ng/ml SCF, 100 ng/ml TPO, and 100 ng/ml Flt3L was added and the plate was incubated at 37°C.

### CD123 and CCR5 gene editing with CRISPR/Cas9 and rAAV6 virus

#### CD34^+^ HSPC culture condition

Plerixafor-mobilized peripheral blood CD34^+^ HSPCs were purchased from AllCells. The cells were thawed as per the manufacturer’s instructions and cultured at 37°C, 5% CO_2_, and 5% O_2_. Cell culture medium was GMP stem cell growth medium (SCGM) medium from CellGenix supplemented with human cytokine (PeproTech) cocktail containing SCF 100 ng/ml, TPO 100 ng/ml, Flt3L 100 ng/ml, and IL-6 100 ng/ml. UM171 (35 nM; StemCell Technologies), streptomycin (20 mg/ml), and penicillin (20 U/ml) were added into the cell culture medium.

#### CD123 and CCR5 gene editing procedure

HSPCs were cultured and prestimulated for 72 h after thaw and before gene editing. Chemically modified CD123 sgRNA and CCR5 sgRNA were synthesized by Synthego Corporation. SpyFi Cas9 was purchased from Aldevron, LLC. CD123-E51K and CD123-E51T donor rAAV6 virus were purchased from SignaGen Laboratories. CCR5-KO donor rAAV6 virus was produced in HEK-293T cells and purified with AAVpro Purification Kit (TakaRa). Electroporation of the RNP complex was performed using the Lonza 4D-Nucleofector (Lonza Group Ltd.) in P3 Primary Cell Solution with program DZ-100. Donor rAAV6 virus was immediately dispensed onto electroporated cells at a multiplicity of infection (MOI) of 2.5 × 10^3^ vector genomes per cell for CD123 and 5.0 × 10^3^ vector genomes per cell for CCR5 based on the titers determined by droplet digital PCR. The cells were then divided into two halves at 2.5 × 10^5^ cells/ml. One half was plated in SCGM medium supplemented with cytokines and 10 ng/ml IL-3 (PeproTech) as +IL-3 treatment. The other half was plated in SCGM medium supplemented with cytokines only as −IL-3 treatment. After incubation for 24 h, a medium change was performed to remove residual rAAV6 virus. The CD34^+^ HSPCs were cultured for up to 8 d for quantification of gene editing events, CFU assays, and pSTAT5 staining and FACS analysis.

### Methylcellulose CFU assay of non-virally edited HSPCs

CFU assay was started at 72 h after editing. For each condition, 1.1 ml of semi-solid methylcellulose medium (StemCell Technologies) containing 500 cells was plated in a well of a SmartDish (StemCell Technologies) in duplicates. The cells were incubated at 37°C, 5% O_2_, and 5% CO_2_ for 14 d. The resulting progenitor colonies were counted and scored with STEMVision analysis (StemCell Technologies) as per the manufacturer’s instruction. Colonies were picked and subjected to sequencing. Shortly, colonies were washed with PBS and then resuspended in 25 µl DNA-QuickExtract solution. PCRs were performed and sent for Sanger sequencing.

### Methylcellulose CFU assay of AAV-edited HSPCs

CFU assay was started at 48 h after gene editing. For each condition, 1.1 ml of semi-solid methylcellulose medium (StemCell Technologies) containing 300 cells and with 10 ng/ml IL-3 (+IL-3 treatment) or without IL-3 (−IL-3 treatment) were plated in a well of a SmartDish (StemCell Technologies) in duplicates. The cells were incubated at 37°C, 5% O_2_, and 5% CO_2_ for 14 d. The resulting progenitor colonies were counted and scored with STEMVision analysis (StemCell Technologies) as per the manufacturer’s instruction.

### pSTAT5 staining of non-virally edited HSPCs

On day 3 after gene editing, all cells were stimulated with 10 ng/ml IL-3 for 1 h at 37°C and were then subjected to pSTAT5 and CD123 staining. Briefly, after 1 h incubation, cells were fixed in 4% paraformaldehyde and then were permeabilized with ice-cold methanol. The permeabilized cells were stained with Alexa 647 mouse-anti-human Stat5 (pY694), BV650 mouse-anti-human CD123 clone 6H6 antibody, and Alexa 488 MIRG123 in FACS buffer at RT for 1 h. After staining, cells were washed with FACS buffer and then subjected for FACS analysis to Fortessa.

### pSTAT5 staining of AAV-edited HSPCs

On day 8 after gene editing, all cells were switched to growth medium without IL-3 for IL-3 starvation. After 1 d IL-3 starvation, for +IL-3 treatment, 10 ng/ml IL-3 was added back to the cells that were originally cultured in +IL-3 medium. For −IL-3 treatment, IL-3 was not added into the cells that were originally cultured in −IL-3 medium. All cell cultures were incubated for 1 h and then were subjected to pSTAT5 staining. Briefly, after 1 h incubation, cells were lysed and fixed in BD Phosflow Lyse/Fix Buffer and then permeabilized with BD Phosflow Perm Buffer III (BD Biosciences) following the manufacturer’s instruction. The permeabilized cells were stained with Alexa 647 Mouse-anti-human Stat5 (pY694) antibody in PBS at RT for 1 h. Alexa 647 Mouse isotype IgG (BD Pharmingen) was used as a negative control. K-562 cells that were not subjected to IL-3 treatment or IL-3 stimulation were stained with the same pSTAT5 antibody as a positive control. After staining, cells were washed with PBS and then subjected to CytoFLEX Flow Cytometer (Beckman Coulter Life Sciences) for FACS analysis.

### Differentiation of non-virally edited HSPCs in vitro

3 d after EP, cells were resuspended in StemPro media (Gibco) containing StemPro Nutrients, low density lipoprotein 50 ng/ml, P/S 1%, glutamine 1%, Flt3 20 ng/ml, TPO 50 ng/ml, IL-6 50 ng/ml, IL-3 10 ng/ml, IL-2 10 ng/ml, IL-7 20 ng/ml, erythropoietin 3 ng/ml, GM-CSF 20 ng/ml, and SCF 100 ng/ml, and 2,000 cells/well were plated in a round bottom 96-well plate. In some wells, CSL362-ADC at a concentration of 10 ng/ml was added. After 14 d cells were collected, stained for CD33, GlyA/CD235a, CD14, CD15, and CD123, and acquired on a Fortessa.

### Mice

All animal work was performed in accordance with the federal and cantonal laws of Switzerland. Protocols were approved by the Animal Research Commission of the Canton of Basel-Stadt, Switzerland. All mice were housed in a specific pathogen–free condition in accordance with institutional guidelines and ethical regulations. NBSGW (stock# 026622) female mice were purchased from Jackson Laboratories. The HSPCs were edited as described above. 2 d after EP, cells were collected and frozen in CS10 (Stem Cell Technologies). Cells were thawed on the day of injection, washed, and resuspended in PBS at 10 × 10^6^ live cells/ml. Recipient NBSGW female mice (4 wk old) were injected into the tail vein. Chimerism was analyzed after 6 and 10 wk in the blood by flow cytometry. Mice were euthanized 16 wk after humanization.

For secondary transplant, NSG-SGM3 (stock#013062) female mice were purchased from Jackson Laboratories. Mice were irradiated the day before BM transplant with 200 cGy. Primary transplant mice were euthanized, bone marrow was isolated, and half of the bone marrow was reinjected into the new host. Mice from secondary transplant were euthanized 8 wk after transplant.

### In vitro TCE-mediated killing assay of non-virally engineered HSPCs

For the TCE killing assays, the HSPCs were edited as described above. Autologous human T cells were isolated from PBMCs as abovementioned and cultured overnight in supplemented media without stimulation. 2 d after EP, edited HSPCs were co-cultured in a 96 U-bottom plate together with human effector T cells at an effector to target ratio of 3:1 and the CD3/CSL362-TCE at 100 ng/ml for 72 h at 37°C. In a set of experiments, MOLM-14 (labeled with CTV according to the manufacturer’s instruction) was added to the autologous T cells and edited HSPCs at an effector to target to tumor ratio of 3:0.5:0.5 together with the CD3/CSL362-TCE (100 ng/ml). Cytotoxic activity (specific killing and elimination of non-edited HSCs) was analyzed by flow cytometry. Specific killing was calculated as follows: (1 − no. alive target cells with TCE/no. alive target cells without TCE) × 100.

### Computational off-target prediction

The Cas-OFFinder algorithm (Bae et al., 2014; release 2.4.1) was used to search the human reference genome hg38 for potential off-target sites in silico using the following parameters: maximum number of mismatches = 3; maximum number of DNA bulges = 1; maximum number of RNA bulges = 1; and PAM = NGG. The human genome reference hg38 (GRCh38 Genome Reference Consortium Human Reference 38) was downloaded from the Golden Path repository at https://hgdownload.cse.ucsc.edu/goldenPath/hg38/chromosomes/. All chromosomes were used in the alignments. To account for redundant off-target sites, unique target regions were selected by grouping overlapping sites using bedtools cluster (v2.30.0) and selecting a representative alignment. The same off-target analysis was performed for two additional gRNAs targeting the VEGFA site 2 (Chaudhari et al., 2020) and HBB (DeWitt et al., 2016; Wienert et al., 2019) genes, which served as benchmarks.

### Discover-Seq

Cells were nucleofected with an RNP complex to target either CD123 or BFP (used as a negative control) as described above. 13 h after nucleofection, an aliquot of nucleofected cells was transferred to new media and genomic DNA was extracted 5 d later to check for genome editing efficiency. 10 million nucleofected cells were fixed in 1% formaldehyde at RT for 15 min. The fixation reaction was quenched with glycine to a final concentration of 125 mM. Cells were harvested and washed twice with chilled PBS and pellets were snap-frozen and stored at −80°C until processing. To process, cell pellets were thawed on ice and incubated with lysis buffer (LB) 1 (50 mM Hepes–KOH, pH7.5; 140 mM NaCl; 1 mM EDTA; 10% glycerol; 0.5% NP-40 or Igepal CA-630; 0.25% Triton X-100; 1× protease inhibitors) on ice for 10 min. Cells were pelleted by centrifugation and incubated in LB 2 (10 mM Tris–HCL, pH8.0; 200 mM NaCl; 1 mM EDTA; 0.5 mM EDTA; 1× protease inhibitors) for 5 min on ice. The extracted nuclei were pelleted by centrifugation and resuspended in LB 3 (10 mM Tris–HCl, pH8; 100 mM NaCl; 1 mM EDTA; 0.1% NaDeoxycholate; 0.5% N-lauroylsarcosine; 1× protease inhibitors). Nuclei were sonicated using a Covaris S2 sonicator with the following settings: duty cycle 5%, intensity 5, 200 cycles per burst, 7 min. Debris was pelleted by centrifugation at 4°C and the supernatant was transferred to a 5-ml tube. 100 μl of Dynabeads protein A that had been prebound with MRE11 antibody (NB 100-142; Novus) were added to the cell lysate and samples were incubated at 4°C overnight with rotation. Beads were collected on a magnetic stand and washed with ice-cold radioimmunoprecipitation assay buffer six times, followed by a final wash with TBS before resuspending beads in 200 µl of elution buffer (50 mM Tris–HCl, pH8; 10 mM EDTA; 1% SDS). The bead slurries were incubated overnight at 65°C to reverse crosslinks. Samples were treated with 1 mg/ml RNaseA (catalog 2271; Ambion) for 30 min at 37°C, followed by proteinase K treatment 20 mg/ml (catalog 25530-049; Invitrogen) for 1 h at 55°C. DNA was then purified using a MinElute PCR Purification Kit (catalog #28004; Qiagen), and sequencing libraries were prepared using a NEBNext Ultra II kit (catalog E7645L; NEB). Samples were sequenced with 50-bp paired-end reads on an Illumina NextSeq at a depth of 30 million reads per sample. Following sequencing, data were analyzed using the BLENDER2 pipeline (available on GitHub).

### rhAmpSeq

Validation of off-target sites was performed using the rhAmpSeq system from IDT. rhAmpSeq primer panels for targeted amplification were generated using the rhAmpSeq design tool defining the insert size between 150 and 250 bp. Applied primer sequences are listed in Table S4 A. rhAmpSeq CRISPR library was prepared according to the manufacturer’s instructions and sequenced on an Illumina MiniSeq instrument (MiniSeq Mid Output Kit, 300-cycles). Sequencing data were analyzed with the rhAmpSeq CRISPR Analysis tool from IDT using the default settings.

### CAST-Seq

Cells were nucleofected with RNP complexes to target CD123. Genomic DNA was extracted 5 d later using the NucleoSpin Tissue kit (Machery and Nagel). CAST-Seq library preparations were performed as described (Turchiano et al., 2021), and data were analyzed using an improved bioinformatics pipeline (Rhiel et al., 2023). Applied primer sequences are listed in Table S3.

### Statistical analysis

Statistical analysis was performed on Prism 9.1.2 software (GraphPad). The number of donors and replicates are found within each figure legend. For multiple comparisons, two-way ANOVA tests were used, with significance levels indicated as follows: not significant (ns) P > 0.05, *P ≤ 0.05, **P ≤ 0.01, ***P ≤ 0.001, and ****P ≤ 0.0001. Data are presented as mean ± standard deviation (SD).

### Online supplemental material

Fig. S1 shows TCE-mediated activation among effector T cell subsets, and Fig. S2 illustrates 123CAR design and production, as well as the activation status of 123CAR T cell subsets after co-culture with wt CD123 and its variants. Both Figs. S1 and S2 complement Fig. 3. Fig. S3 amends Fig. 5 and indicates the non-viral editing strategy for CD123 variants in TF-1 cells and HSPCs, as well as the gating strategy for CD123 expression in phenotypic long-term LT-HSCs, and multipotent progenitors MPP1 and MPP2. This figure also includes data on in vitro IL-3 signaling and differentiation potential in AAV6-edited HSPCs. Fig. S4 is supplementary to Fig. 6 and shows the engraftment of edited HSPCs in peripheral blood and the gating strategy to identify pDCs in the spleen. Fig. S5 adds evidence on tumor-selective CD123-targeted immunotherapy in co-culture assays with MOLM-14 in vitro and is related to Fig. 7. Table S1 includes off-target sites of the CD123-targeting gRNA based on computational prediction. Table S2, A and B, lists Discover-Seq data. Table S3 indicates sequences used for CAST-Seq. Tables S1, S2, and S3 are related to Fig. 5. Table S4 specifies all sequences of crRNAs, HDRT, primers (Table S4 A), and flow cytometry antibodies (Table S4 B) used in the study.

## Supplementary Material

Table S1 includes off-target sites of the CD123-targeting gRNA based on computational prediction.

Table S2 lists Discover-Seq data.

Table S3 indicates sequences used for CAST-Seq.

Table S4 specifies all sequences of crRNAs, HDRT, primers (A) and flow cytometry antibodies (B) used in the study.

SourceData FS2 contains original blots for Fig. S2.

## Data Availability

Datasets are available from the corresponding author upon reasonable request.

## References

[bib1] Anzalone, A.V., L.W. Koblan, and D.R. Liu. 2020. Genome editing with CRISPR-Cas nucleases, base editors, transposases and prime editors. Nat. Biotechnol. 38:824–844. 10.1038/s41587-020-0561-9 32572269

[bib2] Arai, Y., U. Choi, C.I. Corsino, S.M. Koontz, M. Tajima, C.L. Sweeney, M.A. Black, S.A. Feldman, M.C. Dinauer, and H.L. Malech. 2018. Myeloid conditioning with c-kit-Targeted CAR-T cells enables donor stem cell engraftment. Mol. Ther. 26:1181–1197. 10.1016/j.ymthe.2018.03.003 29622475 PMC5993968

[bib3] Asano, T., B. Boisson, F. Onodi, D. Matuozzo, M. Moncada-Velez, M.R.L. Maglorius Renkilaraj, P. Zhang, L. Meertens, A. Bolze, M. Materna, . 2021. X-linked recessive TLR7 deficiency in ∼1% of men under 60 years old with life-threatening COVID-19. Sci. Immunol. 6:eabl4348. 10.1126/sciimmunol.abl4348 34413140 PMC8532080

[bib4] Bae, S., J. Park, and J.S. Kim. 2014. Cas-OFFinder: A fast and versatile algorithm that searches for potential off-target sites of Cas9 RNA-guided endonucleases. Bioinformatics. 30:1473–1475. 10.1093/bioinformatics/btu048 24463181 PMC4016707

[bib5] Baroni, M.L., D. Sanchez Martinez, F. Gutierrez Aguera, H. Roca Ho, M. Castella, S.R. Zanetti, T. Velasco Hernandez, R. Diaz de la Guardia, J. Castano, E. Anguita, . 2020. 41BB-based and CD28-based CD123-redirected T-cells ablate human normal hematopoiesis in vivo. J. Immunother. Cancer. 8:e000845. 10.1136/jitc-2020-000845 32527933 PMC7292050

[bib6] Borot, F., H. Wang, Y. Ma, T. Jafarov, A. Raza, A.M. Ali, and S. Mukherjee. 2019. Gene-edited stem cells enable CD33-directed immune therapy for myeloid malignancies. Proc. Natl. Acad. Sci. USA. 116:11978–11987. 10.1073/pnas.1819992116 31138698 PMC6575599

[bib7] Brentjens, R.J., I. Rivière, J.H. Park, M.L. Davila, X. Wang, J. Stefanski, C. Taylor, R. Yeh, S. Bartido, O. Borquez-Ojeda, . 2011. Safety and persistence of adoptively transferred autologous CD19-targeted T cells in patients with relapsed or chemotherapy refractory B-cell leukemias. Blood. 118:4817–4828. 10.1182/blood-2011-04-348540 21849486 PMC3208293

[bib8] Broughton, S.E., T.R. Hercus, M.P. Hardy, B.J. McClure, T.L. Nero, M. Dottore, H. Huynh, H. Braley, E.F. Barry, W.L. Kan, . 2014. Dual mechanism of interleukin-3 receptor blockade by an anti-cancer antibody. Cell Rep. 8:410–419. 10.1016/j.celrep.2014.06.038 25043189

[bib9] Broughton, S.E., T.R. Hercus, T.L. Nero, W.L. Kan, E.F. Barry, M. Dottore, K.S. Cheung Tung Shing, C.J. Morton, U. Dhagat, M.P. Hardy, . 2018. A dual role for the N-terminal domain of the IL-3 receptor in cell signalling. Nat. Commun. 9:386. 10.1038/s41467-017-02633-7 29374162 PMC5785977

[bib10] Busfield, S.J., M. Biondo, M. Wong, H.S. Ramshaw, E.M. Lee, S. Ghosh, H. Braley, C. Panousis, A.W. Roberts, S.Z. He, . 2014. Targeting of acute myeloid leukemia in vitro and in vivo with an anti-CD123 mAb engineered for optimal ADCC. Leukemia. 28:2213–2221. 10.1038/leu.2014.128 24705479

[bib11] Carter, P.J., and A. Rajpal. 2022. Designing antibodies as therapeutics. Cell. 185:2789–2805. 10.1016/j.cell.2022.05.029 35868279

[bib12] Chaudhari, H.G., J. Penterman, H.J. Whitton, S.J. Spencer, N. Flanagan, M.C. Lei Zhang, E. Huang, A.S. Khedkar, J.M. Toomey, C.A. Shearer, . 2020. Evaluation of homology-independent CRISPR-Cas9 off-target assessment methods. CRISPR J. 3:440–453. 10.1089/crispr.2020.0053 33346710 PMC7757695

[bib13] Chichili, G.R., L. Huang, H. Li, S. Burke, L. He, Q. Tang, L. Jin, S. Gorlatov, V. Ciccarone, F. Chen, . 2015. A CD3xCD123 bispecific DART for redirecting host T cells to myelogenous leukemia: Preclinical activity and safety in nonhuman primates. Sci. Transl. Med. 7:289ra82. 10.1126/scitranslmed.aaa5693 26019218

[bib14] Chiesa, R., C. Georgiadis, F. Syed, H. Zhan, A. Etuk, S.A. Gkazi, R. Preece, G. Ottaviano, T. Braybrook, J. Chu, . 2023. Base-Edited CAR7 T Cells for Relapsed T-Cell Acute Lymphoblastic Leukemia. N. Engl. J. Med. 389:899–910. 10.1056/NEJMoa2300709 37314354

[bib15] Clement, K., H. Rees, M.C. Canver, J.M. Gehrke, R. Farouni, J.Y. Hsu, M.A. Cole, D.R. Liu, J.K. Joung, D.E. Bauer, and L. Pinello. 2019. CRISPResso2 provides accurate and rapid genome editing sequence analysis. Nat. Biotechnol. 37:224–226. 10.1038/s41587-019-0032-3 30809026 PMC6533916

[bib16] Cromer, M.K., V.V. Barsan, E. Jaeger, M. Wang, J.P. Hampton, F. Chen, D. Kennedy, J. Xiao, I. Khrebtukova, A. Granat, . 2022. Ultra-deep sequencing validates safety of CRISPR/Cas9 genome editing in human hematopoietic stem and progenitor cells. Nat. Commun. 13:4724. 10.1038/s41467-022-32233-z 35953477 PMC9372057

[bib17] Czechowicz, A., D. Kraft, I.L. Weissman, and D. Bhattacharya. 2007. Efficient transplantation via antibody-based clearance of hematopoietic stem cell niches. Science. 318:1296–1299. 10.1126/science.1149726 18033883 PMC2527021

[bib18] Czechowicz, A., R. Palchaudhuri, A. Scheck, Y. Hu, J. Hoggatt, B. Saez, W.W. Pang, M.K. Mansour, T.A. Tate, Y.Y. Chan, . 2019. Selective hematopoietic stem cell ablation using CD117-antibody-drug-conjugates enables safe and effective transplantation with immunity preservation. Nat. Commun. 10:617. 10.1038/s41467-018-08201-x 30728354 PMC6365495

[bib19] Dever, D.P., R.O. Bak, A. Reinisch, J. Camarena, G. Washington, C.E. Nicolas, M. Pavel-Dinu, N. Saxena, A.B. Wilkens, S. Mantri, . 2016. CRISPR/Cas9 β-globin gene targeting in human haematopoietic stem cells. Nature. 539:384–389. 10.1038/nature20134 27820943 PMC5898607

[bib20] DeWitt, M.A., W. Magis, N.L. Bray, T. Wang, J.R. Berman, F. Urbinati, S.J. Heo, T. Mitros, D.P. Munoz, D. Boffelli, . 2016. Selection-free genome editing of the sickle mutation in human adult hematopoietic stem/progenitor cells. Sci. Transl. Med. 8:360ra134. 10.1126/scitranslmed.aaf9336 PMC5500303 27733558

[bib21] Dunn, S.D., L.M. Wahl, and G.B. Gloor. 2008. Mutual information without the influence of phylogeny or entropy dramatically improves residue contact prediction. Bioinformatics. 24:333–340. 10.1093/bioinformatics/btm604 18057019

[bib22] Ekeberg, M., C. Lövkvist, Y. Lan, M. Weigt, and E. Aurell. 2013. Improved contact prediction in proteins: Using pseudolikelihoods to infer potts models. Phys. Rev. E Stat. Nonlin. Soft Matter Phys. 87:012707. 10.1103/PhysRevE.87.012707 23410359

[bib23] Emerson, S.G., Y.C. Yang, S.C. Clark, and M.W. Long. 1988. Human recombinant granulocyte-macrophage colony stimulating factor and interleukin 3 have overlapping but distinct hematopoietic activities. J. Clin. Invest. 82:1282–1287. 10.1172/JCI113727 3049674 PMC442680

[bib24] Everette, K.A., G.A. Newby, R.M. Levine, K. Mayberry, Y. Jang, T. Mayuranathan, N. Nimmagadda, E. Dempsey, Y. Li, S.V. Bhoopalan, . 2023. Ex vivo prime editing of patient haematopoietic stem cells rescues sickle-cell disease phenotypes after engraftment in mice. Nat. Biomed. Eng. 7:616–628. 10.1038/s41551-023-01026-0 37069266 PMC10195679

[bib25] Eyquem, J., J. Mansilla-Soto, T. Giavridis, S.J. van der Stegen, M. Hamieh, K.M. Cunanan, A. Odak, M. Gönen, and M. Sadelain. 2017. Targeting a CAR to the TRAC locus with CRISPR/Cas9 enhances tumour rejection. Nature. 543:113–117. 10.1038/nature21405 28225754 PMC5558614

[bib26] Finck, A.V., T. Blanchard, C.P. Roselle, G. Golinelli, and C.H. June. 2022. Engineered cellular immunotherapies in cancer and beyond. Nat. Med. 28:678–689. 10.1038/s41591-022-01765-8 35440724 PMC9305718

[bib27] Gill, S., S.K. Tasian, M. Ruella, O. Shestova, Y. Li, D.L. Porter, M. Carroll, G. Danet-Desnoyers, J. Scholler, S.A. Grupp, . 2014. Preclinical targeting of human acute myeloid leukemia and myeloablation using chimeric antigen receptor-modified T cells. Blood. 123:2343–2354. 10.1182/blood-2013-09-529537 24596416 PMC3983612

[bib28] Globerson Levin, A., I. Rivière, Z. Eshhar, and M. Sadelain. 2021. CAR T cells: Building on the CD19 paradigm. Eur. J. Immunol. 51:2151–2163. 10.1002/eji.202049064 34196410 PMC9392049

[bib29] Haubner, S., F. Perna, T. Köhnke, C. Schmidt, S. Berman, C. Augsberger, F.M. Schnorfeil, C. Krupka, F.S. Lichtenegger, X. Liu, . 2019. Coexpression profile of leukemic stem cell markers for combinatorial targeted therapy in AML. Leukemia. 33:64–74. 10.1038/s41375-018-0180-3 29946192 PMC6326956

[bib30] Hopf, T.A., J.B. Ingraham, F.J. Poelwijk, C.P. Schärfe, M. Springer, C. Sander, and D.S. Marks. 2017. Mutation effects predicted from sequence co-variation. Nat. Biotechnol. 35:128–135. 10.1038/nbt.3769 28092658 PMC5383098

[bib31] Hopf, T.A., A.G. Green, B. Schubert, S. Mersmann, C.P.I. Schärfe, J.B. Ingraham, A. Toth-Petroczy, K. Brock, A.J. Riesselman, P. Palmedo, . 2019. The EVcouplings python framework for coevolutionary sequence analysis. Bioinformatics. 35:1582–1584. 10.1093/bioinformatics/bty862 30304492 PMC6499242

[bib32] Humbert, O., G.S. Laszlo, S. Sichel, C. Ironside, K.G. Haworth, O.M. Bates, M.E. Beddoe, R.R. Carrillo, H.P. Kiem, and R.B. Walter. 2019. Engineering resistance to CD33-targeted immunotherapy in normal hematopoiesis by CRISPR/Cas9-deletion of CD33 exon 2. Leukemia. 33:762–808. 10.1038/s41375-018-0277-8 30291334

[bib33] Hutmacher, C., L. Volta, F. Rinaldi, P. Murer, R. Myburgh, M.G. Manz, and D. Neri. 2019. Development of a novel fully-human anti-CD123 antibody to target acute myeloid leukemia. Leuk. Res. 84:106178. 10.1016/j.leukres.2019.106178 31326578

[bib34] Irving, M., V. Zoete, M. Bassani-Sternberg, and G. Coukos. 2022. A roadmap for driving CAR T cells toward the oncogenic immunopeptidome. Cancer Cell. 40:20–22. 10.1016/j.ccell.2021.12.011 35016027

[bib35] Jordan, C.T., D. Upchurch, S.J. Szilvassy, M.L. Guzman, D.S. Howard, A.L. Pettigrew, T. Meyerrose, R. Rossi, B. Grimes, D.A. Rizzieri, . 2000. The interleukin-3 receptor alpha chain is a unique marker for human acute myelogenous leukemia stem cells. Leukemia. 14:1777–1784. 10.1038/sj.leu.2401903 11021753

[bib36] June, C.H., R.S. O’Connor, O.U. Kawalekar, S. Ghassemi, and M.C. Milone. 2018. CAR T cell immunotherapy for human cancer. Science. 359:1361–1365. 10.1126/science.aar6711 29567707

[bib37] Kalos, M., B.L. Levine, D.L. Porter, S. Katz, S.A. Grupp, A. Bagg, and C.H. June. 2011. T cells with chimeric antigen receptors have potent antitumor effects and can establish memory in patients with advanced leukemia. Sci. Transl. Med. 3:95ra73. 10.1126/scitranslmed.3002842 PMC3393096 21832238

[bib38] Kim, M.Y., K.R. Yu, S.S. Kenderian, M. Ruella, S. Chen, T.H. Shin, A.A. Aljanahi, D. Schreeder, M. Klichinsky, O. Shestova, . 2018. Genetic inactivation of CD33 in hematopoietic stem cells to enable CAR T cell immunotherapy for acute myeloid leukemia. Cell. 173:1439–1453.e19. 10.1016/j.cell.2018.05.013 29856956 PMC6003425

[bib39] Kim, L., B. Fowler, C.M. Campbell, J. Slivnick, H. Nawaz, Y. Kaka, P. Ruz, A. Vallakati, R. Baliga, S. Vasu, and D. Addison. 2021. Acute cardiotoxicity after initiation of the novel tyrosine kinase inhibitor gilteritinib for acute myeloid leukemia. Cardiooncology. 7:36. 10.1186/s40959-021-00122-x 34686212 PMC8531894

[bib40] Kitamura, T., T. Tange, T. Terasawa, S. Chiba, T. Kuwaki, K. Miyagawa, Y.F. Piao, K. Miyazono, A. Urabe, and F. Takaku. 1989. Establishment and characterization of a unique human cell line that proliferates dependently on GM-CSF, IL-3, or erythropoietin. J. Cell. Physiol. 140:323–334. 10.1002/jcp.1041400219 2663885

[bib41] Kochenderfer, J.N., W.H. Wilson, J.E. Janik, M.E. Dudley, M. Stetler-Stevenson, S.A. Feldman, I. Maric, M. Raffeld, D.A. Nathan, B.J. Lanier, . 2010. Eradication of B-lineage cells and regression of lymphoma in a patient treated with autologous T cells genetically engineered to recognize CD19. Blood. 116:4099–4102. 10.1182/blood-2010-04-281931 20668228 PMC2993617

[bib42] Kornete, M., R. Marone, and L.T. Jeker. 2018. Highly efficient and versatile plasmid-based gene editing in primary T cells. J. Immunol. 200:2489–2501. 10.4049/jimmunol.1701121 29445007 PMC5857648

[bib43] Kovtun, Y., G.E. Jones, S. Adams, L. Harvey, C.A. Audette, A. Wilhelm, C. Bai, L. Rui, R. Laleau, F. Liu, . 2018. A CD123-targeting antibody-drug conjugate, IMGN632, designed to eradicate AML while sparing normal bone marrow cells. Blood Adv. 2:848–858. 10.1182/bloodadvances.2018017517 29661755 PMC5916008

[bib44] Larrue, C., S. Mouche, S. Lin, F. Simonetta, N.K. Scheidegger, L. Poulain, R. Birsen, J.E. Sarry, K. Stegmaier, and J. Tamburini. 2023. Mitochondrial fusion is a therapeutic vulnerability of acute myeloid leukemia. Leukemia. 37:765–775. 10.1038/s41375-023-01835-x 36739349 PMC10079528

[bib45] Lee, B., and F.M. Richards. 1971. The interpretation of protein structures: Estimation of static accessibility. J. Mol. Biol. 55:379–400. 10.1016/0022-2836(71)90324-X 5551392

[bib46] Lee, D.W., J.N. Kochenderfer, M. Stetler-Stevenson, Y.K. Cui, C. Delbrook, S.A. Feldman, T.J. Fry, R. Orentas, M. Sabatino, N.N. Shah, . 2015. T cells expressing CD19 chimeric antigen receptors for acute lymphoblastic leukaemia in children and young adults: A phase 1 dose-escalation trial. Lancet. 385:517–528. 10.1016/S0140-6736(14)61403-3 25319501 PMC7065359

[bib47] Li, Z., A. Czechowicz, A. Scheck, D.J. Rossi, and P.M. Murphy. 2019. Hematopoietic chimerism and donor-specific skin allograft tolerance after non-genotoxic CD117 antibody-drug-conjugate conditioning in MHC-mismatched allotransplantation. Nat. Commun. 10:616. 10.1038/s41467-018-08202-w 30728353 PMC6365540

[bib48] Liu, T.F., J.O. Urieto, J.E. Moore, M.S. Miller, A.C. Lowe, A. Thorburn, and A.E. Frankel. 2004. Diphtheria toxin fused to variant interleukin-3 provides enhanced binding to the interleukin-3 receptor and more potent leukemia cell cytotoxicity. Exp. Hematol. 32:277–281. 10.1016/j.exphem.2003.11.010 15003313

[bib49] Lynn, R.C., E.W. Weber, E. Sotillo, D. Gennert, P. Xu, Z. Good, H. Anbunathan, J. Lattin, R. Jones, V. Tieu, . 2019. c-Jun overexpression in CAR T cells induces exhaustion resistance. Nature. 576:293–300. 10.1038/s41586-019-1805-z 31802004 PMC6944329

[bib50] Majzner, R.G., and C.L. Mackall. 2018. Tumor antigen escape from CAR T-cell therapy. Cancer Discov. 8:1219–1226. 10.1158/2159-8290.CD-18-0442 30135176

[bib51] Majzner, R.G., and C.L. Mackall. 2019. Clinical lessons learned from the first leg of the CAR T cell journey. Nat. Med. 25:1341–1355. 10.1038/s41591-019-0564-6 31501612

[bib52] Mallampati, S., B. Sun, Y. Lu, H. Ma, Y. Gong, D. Wang, J.S. Lee, K. Lin, and X. Sun. 2014. Integrated genetic approaches identify the molecular mechanisms of Sox4 in early B-cell development: Intricate roles for RAG1/2 and CK1ε. Blood. 123:4064–4076. 10.1182/blood-2013-12-543801 24786772 PMC4073324

[bib53] Maude, S.L., N. Frey, P.A. Shaw, R. Aplenc, D.M. Barrett, N.J. Bunin, A. Chew, V.E. Gonzalez, Z. Zheng, S.F. Lacey, . 2014. Chimeric antigen receptor T cells for sustained remissions in leukemia. N. Engl. J. Med. 371:1507–1517. 10.1056/NEJMoa1407222 25317870 PMC4267531

[bib54] Melenhorst, J.J., G.M. Chen, M. Wang, D.L. Porter, C. Chen, M.A. Collins, P. Gao, S. Bandyopadhyay, H. Sun, Z. Zhao, . 2022. Decade-long leukaemia remissions with persistence of CD4^+^ CAR T cells. Nature. 602:503–509. 10.1038/s41586-021-04390-6 35110735 PMC9166916

[bib55] Mitternacht, S. 2016. FreeSASA: An open source C library for solvent accessible surface area calculations. F1000 Res. 5:189. 10.12688/f1000research.7931.1 PMC4776673 26973785

[bib56] Mougiakakos, D., G. Krönke, S. Völkl, S. Kretschmann, M. Aigner, S. Kharboutli, S. Böltz, B. Manger, A. Mackensen, and G. Schett. 2021. CD19-targeted CAR T cells in refractory systemic lupus erythematosus. N. Engl. J. Med. 385:567–569. 10.1056/NEJMc2107725 34347960

[bib57] Myburgh, R., J.D. Kiefer, N.F. Russkamp, C.F. Magnani, N. Nuñez, A. Simonis, S. Pfister, C.M. Wilk, D. McHugh, J. Friemel, . 2020. Anti-human CD117 CAR T-cells efficiently eliminate healthy and malignant CD117-expressing hematopoietic cells. Leukemia. 34:2688–2703. 10.1038/s41375-020-0818-9 32358567

[bib58] Nguyen, D.N., T.L. Roth, P.J. Li, P.A. Chen, R. Apathy, M.R. Mamedov, L.T. Vo, V.R. Tobin, D. Goodman, E. Shifrut, . 2020. Polymer-stabilized Cas9 nanoparticles and modified repair templates increase genome editing efficiency. Nat. Biotechnol. 38:44–49. 10.1038/s41587-019-0325-6 31819258 PMC6954310

[bib59] Niswander, L.M., Z.T. Graff, C.D. Chien, J.A. Chukinas, C.A. Meadows, L.C. Leach, J.P. Loftus, M.E. Kohler, S.K. Tasian, and T.J. Fry. 2023. Potent preclinical activity of FLT3-directed chimeric antigen receptor T-cell immunotherapy against *FLT3*- mutant acute myeloid leukemia and *KMT2A*-rearranged acute lymphoblastic leukemia. Haematologica. 108:457–471. 10.3324/haematol.2022.281456 35950535 PMC9890025

[bib60] Palchaudhuri, R., B. Saez, J. Hoggatt, A. Schajnovitz, D.B. Sykes, T.A. Tate, A. Czechowicz, Y. Kfoury, F. Ruchika, D.J. Rossi, . 2016. Non-genotoxic conditioning for hematopoietic stem cell transplantation using a hematopoietic-cell-specific internalizing immunotoxin. Nat. Biotechnol. 34:738–745. 10.1038/nbt.3584 27272386 PMC5179034

[bib61] Paszkiewicz, P.J., S.P. Fräßle, S. Srivastava, D. Sommermeyer, M. Hudecek, I. Drexler, M. Sadelain, L. Liu, M.C. Jensen, S.R. Riddell, and D.H. Busch. 2016. Targeted antibody-mediated depletion of murine CD19 CAR T cells permanently reverses B cell aplasia. J. Clin. Invest. 126:4262–4272. 10.1172/JCI84813 27760047 PMC5096899

[bib62] Perna, F., S.H. Berman, R.K. Soni, J. Mansilla-Soto, J. Eyquem, M. Hamieh, R.C. Hendrickson, C.W. Brennan, and M. Sadelain. 2017. Integrating proteomics and transcriptomics for systematic combinatorial chimeric antigen receptor therapy of AML. Cancer Cell. 32:506–519.e5. 10.1016/j.ccell.2017.09.004 29017060 PMC7025434

[bib63] Pfister, O., V. Lorenz, A. Oikonomopoulos, L. Xu, S.P. Häuselmann, C. Mbah, B.A. Kaufmann, R. Liao, A. Wodnar-Filipowicz, and G.M. Kuster. 2014. FLT3 activation improves post-myocardial infarction remodeling involving a cytoprotective effect on cardiomyocytes. J. Am. Coll. Cardiol. 63:1011–1019. 10.1016/j.jacc.2013.08.1647 24184252

[bib64] Porter, D.L., B.L. Levine, M. Kalos, A. Bagg, and C.H. June. 2011. Chimeric antigen receptor-modified T cells in chronic lymphoid leukemia. N. Engl. J. Med. 365:725–733. 10.1056/NEJMoa1103849 21830940 PMC3387277

[bib65] Rhiel, M., K. Geiger, G. Andrieux, J. Rositzka, M. Boerries, T. Cathomen, and T.I. Cornu. 2023. T-CAST: An optimized CAST-seq pipeline for TALEN confirms superior safety and efficacy of obligate-heterodimeric scaffolds. Front. Genome Ed. 5:1130736. 10.3389/fgeed.2023.1130736 36890979 PMC9986454

[bib66] Roberts, A.W., S. He, D. Ritchie, M.S. Hertzberg, I. Kerridge, S.T. Durrant, G. Kennedy, I.D. Lewis, P. Mariton, A.J. McLachlan, and C.R. Dev. 2010. A phase I study of anti-CD123 monocional antibody (mAb) CSL360 targeting leukemia stem cells (LSC) in AML. J. Clin. Oncol. 28:e13012. 10.1200/jco.2010.28.15_suppl.e13012

[bib67] Roth, T.L., P.J. Li, F. Blaeschke, J.F. Nies, R. Apathy, C. Mowery, R. Yu, M.L.T. Nguyen, Y. Lee, A. Truong, . 2020. Pooled knockin targeting for genome engineering of cellular immunotherapies. Cell. 181:728–744.e21. 10.1016/j.cell.2020.03.039 32302591 PMC7219528

[bib68] Roth, T.L., C. Puig-Saus, R. Yu, E. Shifrut, J. Carnevale, P.J. Li, J. Hiatt, J. Saco, P. Krystofinski, H. Li, . 2018. Reprogramming human T cell function and specificity with non-viral genome targeting. Nature. 559:405–409. 10.1038/s41586-018-0326-5 29995861 PMC6239417

[bib69] Sabatier, M., R. Birsen, L. Lauture, S. Mouche, P. Angelino, J. Dehairs, L. Goupille, I. Boussaid, M. Heiblig, E. Boet, . 2023. C/EBPa confers dependence to fatty acid anabolic pathways and vulnerability to lipid oxidative stress-induced ferroptosis in FLT3-mutant leukemia. Cancer Discov. 13:1720–1747. 10.1158/2159-8290.CD-22-0411 37012202

[bib70] Strohl, W.R., and M. Naso. 2019. Bispecific T-cell redirection versus chimeric antigen receptor (CAR)-T cells as approaches to kill cancer cells. Antibodies. 8:41. 10.3390/antib8030041 31544847 PMC6784091

[bib71] Suzek, B.E., Y. Wang, H. Huang, P.B. McGarvey, C.H. Wu, and C. Uniprot. 2015. UniRef clusters: A comprehensive and scalable alternative for improving sequence similarity searches. Bioinformatics. 31:926–932. 10.1093/bioinformatics/btu739 25398609 PMC4375400

[bib72] Tiller, T., E. Meffre, S. Yurasov, M. Tsuiji, M.C. Nussenzweig, and H. Wardemann. 2008. Efficient generation of monoclonal antibodies from single human B cells by single cell RT-PCR and expression vector cloning. J. Immunol. Methods. 329:112–124. 10.1016/j.jim.2007.09.017 17996249 PMC2243222

[bib73] Turchiano, G., G. Andrieux, J. Klermund, G. Blattner, V. Pennucci, M. El Gaz, G. Monaco, S. Poddar, C. Mussolino, T.I. Cornu, . 2021. Quantitative evaluation of chromosomal rearrangements in gene-edited human stem cells by CAST-Seq. Cell Stem Cell. 28:1136–1147.e5. 10.1016/j.stem.2021.02.002 33626327

[bib74] Vander Mause, E.R., D. Atanackovic, C.S. Lim, and T. Luetkens. 2022. Roadmap to affinity-tuned antibodies for enhanced chimeric antigen receptor T cell function and selectivity. Trends Biotechnol. 40:875–890. 10.1016/j.tibtech.2021.12.009 35078657

[bib75] Wienert, B., S.K. Wyman, C.D. Richardson, C.D. Yeh, P. Akcakaya, M.J. Porritt, M. Morlock, J.T. Vu, K.R. Kazane, H.L. Watry, . 2019. Unbiased detection of CRISPR off-targets in vivo using DISCOVER-Seq. Science. 364:286–289. 10.1126/science.aav9023 31000663 PMC6589096

[bib76] Zeng, A.G.X., S. Bansal, L. Jin, A. Mitchell, W.C. Chen, H.A. Abbas, M. Chan-Seng-Yue, V. Voisin, P. van Galen, A. Tierens, . 2022. A cellular hierarchy framework for understanding heterogeneity and predicting drug response in acute myeloid leukemia. Nat. Med. 28:1212–1223. 10.1038/s41591-022-01819-x 35618837

