# Supplementary figures and images for: Epitope-engineered human hematopoietic stem cells are shielded from CD123-targeted immunotherapy
Source: J Exp Med. 2023 Sep 29;220(12):e20231235. doi: 10.1084/jem.20231235 (PMC10541312; doi:10.1084/jem.20231235)

Cropped sections

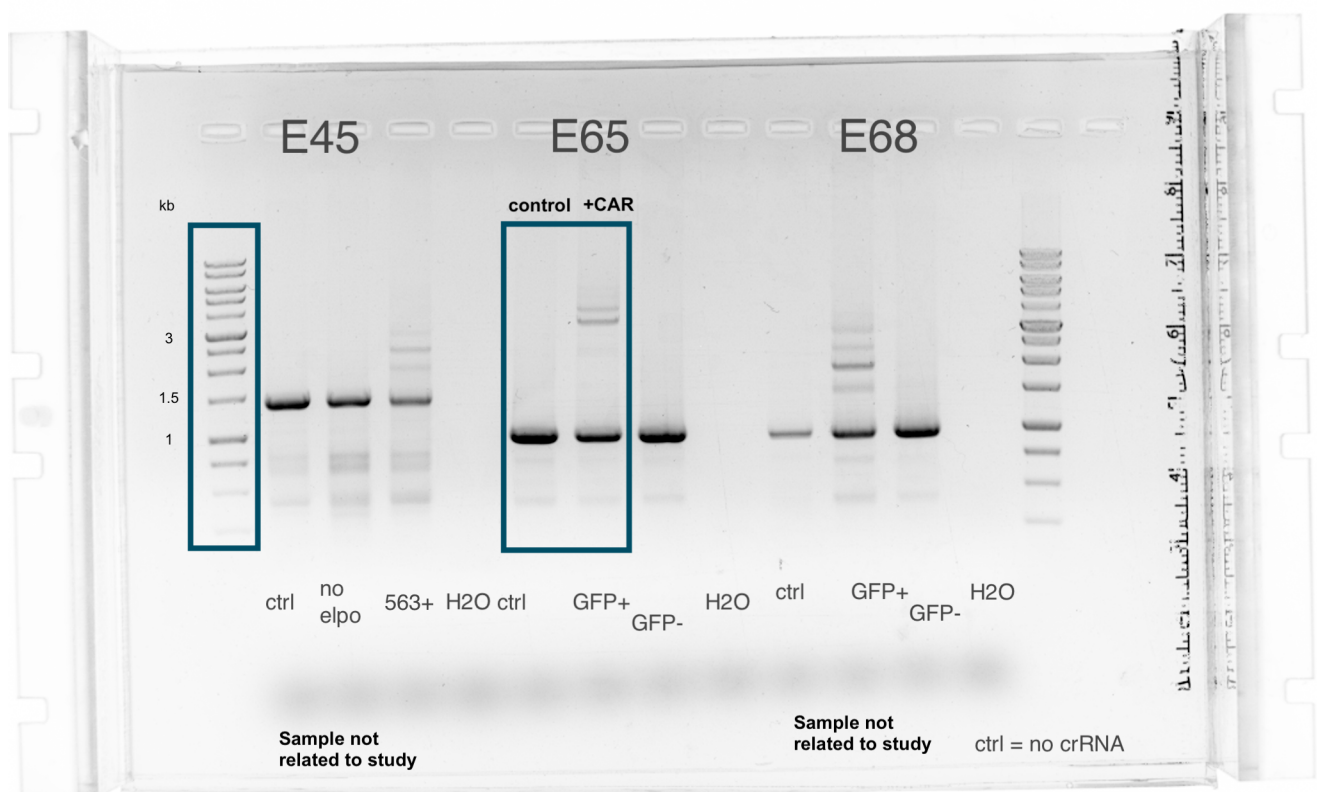

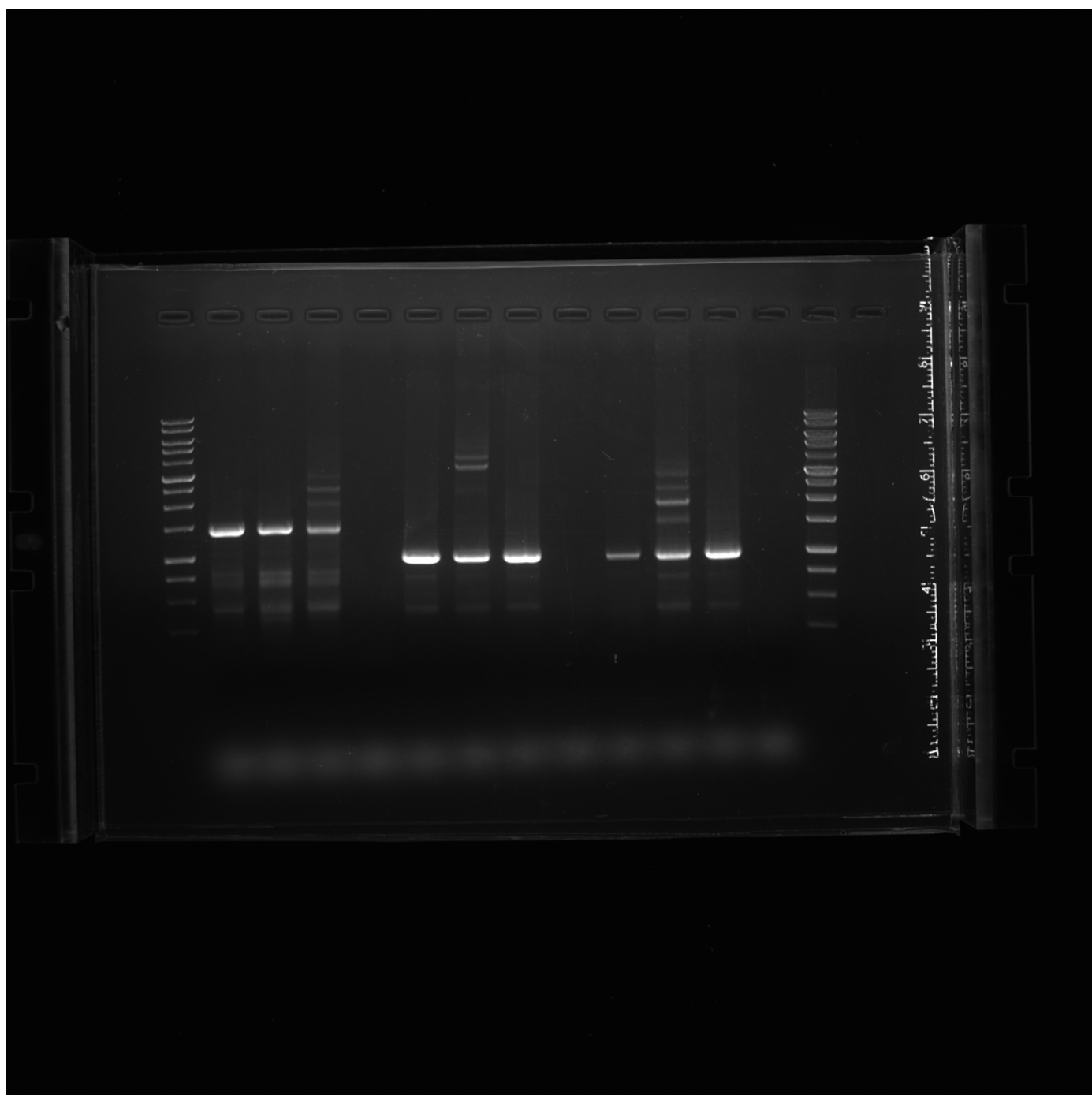

Supplement: SourceData FS2 — contains original blots for Fig. S2. [file JEM_20231235_SourceDataFS2.pdf]
